# A Treatise on Insanity, and Other Disorders Affecting the Mind

**Published:** 1839-01

**Authors:** 


### BRITISH AND FOREIGN MEDICAL REVIEW,

FOR JANUARY, 1839.

### PART FIRST.

### Analytical and Critical Reviews.

ART. I.

1. *A Treatise on Insanity, and other Disorders affecting the Mind.* By JAMES COWLES PRICHARD, M.D. F.R.S., &c. &c.—*London,* 1835. 8vo. pp. 483.

2. *Des Maladies Mentales considérées sous les Rapports Médical, Hygiènique, et Médico-légal.* Par E. ESQUIROL, Médecin en chef de la Maison Royale des Aliénés de Charenton, &c. &c. *Accompagnées de* 27 *Planches gravées.—Paris,* 1838. Tomes ii.—8vo. pp. 676, 864.
*Mental Maladies considered in relation to Medicine, Hygiène, and Medical Jurisprudence.* By E. ESQUIROL, &c. &c.

3. *Essay on the Classification of the Insane.* By M. ALLEN, M.D. &c. —*London, no date.* 8vo. pp. 212; *with Plates.*

4. *A Treatise on the Nature, Symptoms, Causes, and Treatment of Insanity; with Practical Observations on Lunatic Asylums, and a Description of the Pauper Lunatic Asylum for the County of Middlesex, at Hanwell, with a detailed Account of its Management.* By Sir W. C. ELLIS, M.D., Resident Medical Superintendent, and formerly of the Asylum at Wakefield.—*London,* 1838. 8vo. pp. 344.

5. *Delle Malattie della Mente, ovvero delle diverse Specie di Follie.* Opera di LUIGI FERRARESE, Dottor di Medicina, &c.—*Napoli,* 1830 and 1832. Two Vols. 8vo. pp 220, 112.
*Of Maladies of the Mind, or the different Species of Madness.* By LUIGI FERRARESE, M.D. &c.

6. *Tenth Report of the Directors of the Connecticut Retreat for the Insane.*—1834.

7. *Report of the Lunatic Asylum in St. Petersburgh.*—1834.

8. *Saggio sulla Statistica Medica della Real Casa dei Matti di Palermo.* Scritto da ANTONIO GRECO, Medico ordinario della sudetta Real Casa dei Matti, &c.—*Palermo,* 1835. 8vo. pp. 24.
*Medical Statistics of the Royal Institution for the Insane at Palermo.* By A. GRECO, Physician in Ordinary, &c.

9. *State of the New-York Hospital, and Bloomingdale Asylum.*—1835 and 1836.

10. *Sixteenth and Eighteenth Report of the Directors of the Dundee Lunatic Asylum.*—1836 and 1838.

11. *State of an Institution near York, called The Retreat, for Persons afflicted with Disorders of the Mind.*—1836.

12. *Reports and other Documents relating to the State Lunatic Hospital at Worcester, Mass. Printed by order of the Senate.*—1837.

13. *The Forty-fourth Report of the Visiting Justices, and the Seventh Report of the Resident Physician and Treasurer of the Middlesex Pauper Lunatic Asylum.*—1837.

14. *Physician's Report to the Managers of the Lunatic Asylum of Aberdeen.*—1838.

15. *On the Statistics of English Lunatic Asylums, and the Reform of their Public Management.* By WILLIAM FARR.—*London, no date.* 8vo. pp. 46.

16. *Observations on the Management of Madhouses; illustrated by Occurrences in the West-Riding and Middlesex Asylums.* By CALEB CROWTHER, M.D., formerly Senior Physician to the West-Riding Pauper Lunatic Asylum.—*London,* 1838. 8vo. pp. 145.

THE number of publications which have reached us relative to the subject of insanity, since the commencement of the present year, attest that the important class of maladies of which they treat is attracting increased attention; whilst the contents of some of them sufficiently show that there are yet many circumstances requiring better consideration, by which the welfare of lunatics is materially affected. It would require an article extending beyond all reasonable limits to do complete justice to the various treatises and reports of which we have quoted the titles; but we may, perhaps, serviceably attempt to condense and comment upon some of the most important facts and opinions which these numerous works contain.

Independently of the many interesting questions involved in this subject respecting the nature of insanity, and the signs of its actual presence, the practical consideration of its actual management in a mere medicinal sense has scarcely, we think, sufficiently engaged the attention of the profession. Every one acquainted with the details of private practice must know that there are no cases which occasion more perplexity and alarm, and eventually lead to greater practical improprieties, than the various forms of disordered mind. The artificial definitions of mental philosophers have done little more than produce confusion; the demonstrations of morbid anatomists have sometimes shown irremediable changes, and sometimes no changes which satisfactorily explained the phenomena which preceded death; and the practice set forth in books has been often capricious and often empirical. Accustomed to subdue acute diseases by prompt and definite measures, and to control, if not to cure, chronic affections by intelligible indications, the practitioner shuns as much as possible the responsibility of a class of cases in which excitement resists all ordinary methods of reduction, or depression is obstinate under every stimulus; whilst all the offensive peculiarities of lunacy, and the fears of friends, and the accusations of the unfriendly, and various distresses incidental to cases of insanity, combine to render him impatient of his charge, and glad to surrender it, in any way, to any one who will undertake what he deems a thankless, or even a disgusting office.

In a former Number of this Journal we inserted an account of the first public attempt on record to liberate maniacs from chains and dungeons; an act of heroism at the time, as well as of philanthropy, for which Pinel deserved a statue. Openly barbarous plans of treatment are now so uncommon, that a superficial observation might lead to the conclusion that everything was accomplished that humanity could desire or skill could suggest. Numerous establishments exist for the reception of the insane, and many of them are conducted by persons incapable of unnecessary harshness, and only known for their honorable and humane care of those intrusted to them. Some of our public institutions are in certain respects models of excellent management; and successive acts of the legislature have at least had for their object that of throwing efficient protection around the insane of every class of society.

To these admissions we are constrained to add, that the theories of insanity entertained, and occasionally uttered, by medical men are vague and ill considered; that the practice of medicine in mental disorders is unsettled; and that, where the will exists on the part of relatives, much injustice may yet be committed, and with complete impunity. Every one knows how little value is attached to the evidence of medical men in cases of lunacy, on account of the wild and fanciful notions which they often bring into court with them. In common practice, if insanity declares itself, and resists bleeding, blistering, and purging, all the anxiety of the practitioner is to get the patient out of his hands, and to send him no matter where, so that he sees no more of him: and we can ourselves testify, from personal observation, that a nervous patient may be taken from the practitioner who has been in attendance, dragged under the most unfavorable circumstances before another, sentenced to confinement among lunatics, and placed beyond the possible means of immediate liberation. The selfish keeper of a common lunatic-house laughs at attempts to deprive him of a patient. Provided that the letter of the law is complied with as regards the certificates, he cares not how irregularly those certificates have been obtained; and those only who have made a formal remonstrance know with what reluctance the magistrates move, and that the visiting physicians for the county will not stir, and that lord chancellors will not or cannot listen to any appeal. The metropolitan commissioners will be found ready, we sincerely believe, at all times, to do all in their power; but that amounts, in many cases, to nothing: they will, at the utmost, request the magistrates to institute an enquiry; which those magistrates will do, most probably, in presence of the keeper of the lunatic-house, and thus baffle all interference. Such facts have taken place under our own observation, and we fear they are far from uncommon.

When it is also recollected that lunatic asylums were the scenes of incredible barbarity, until the public indignation purged them; when the secrecy with which the proceedings of the commissioners of lunacy are conducted is reflected upon, (necessary, perhaps, in some degree, but open to abuse;) and when it is remembered that the relatives of lunatics seldom hail their restoration to health with gratitude, but, as Mr. Farr expresses it, welcome "the lunatic's return to his senses as ruefully as would a prodigal heir the resurrection of his grandfather;" we find abundant reasons for bringing the subject before the attention of the profession and the public again and again.

Those who feel strongly upon this subject may sometimes be suspected of making exaggerated statements; but let any one look into the past records of York and of Bethlem, and he will find how possible it is for every enormity to be sheltered under the sanction of all that the world calls respectable. These evils were general. The reports of travellers respecting the past state of the asylums of Scotland, of France, of Italy, are appalling; and the documents (obligingly forwarded to us by Dr. Woodward) relative to the past and present condition of the State Lunatic Hospital of Worcester, Massachusetts, give the same testimony of former incredible neglect, now happily superseded by all that skill and humanity can suggest.

M. Esquirol's work is composed of several treatises published at different periods, and now collected into two volumes, in not the best order, as it seems to us; although every treatise and every page published by so able a physician, after forty years' experience, contains something to interest or instruct the medical reader. The section with which the first volume commences, entitled *de la Folie*, comprises numerous facts and statements to which we shall find further occasion to refer. Of those on Hallucinations and Illusions we may observe the same. To these succeed, in the same volume, treatises or essays on Puerperal Insanity, Epilepsy, and Melancholia (*Lypémanie*), Demonomania, and Suicide. In the second volume, M. Esquirol treats of Monomania, Mania, Dementia, and Idiocy; five Statistical and Hygienic Memoirs follow; and three on Insanity considered in its medico-legal relations. Some of these subjects we must necessarily pass over, and others, particularly the one last mentioned, we must defer speaking of until another occasion. Our efforts must at present be confined to selecting a few practical points for such illustration as they derive from the important works before us.

The plan of M. Esquirol's work is more calculated to save trouble to the author than to the reader, and produces some repetitions and an appearance of want of arrangement. Much, if not the whole, of the section *de la Folie* has, we think, been previously published; that on Hallucinations is dated in 1817; that on Illusions in 1832; the one entitled *de la Fureur* in 1816; that on Puerperal Insanity in 1819; that on Epilepsy in 1815; a chapter on the critical terminations of Folie is then inserted, dated in 1818; and the section on Melancholia is dated in 1820. Demonomania and Suicide are dated 1814 and 1821. These subjects being discussed in this inconvenient order in the first volume, the second begins with treatises on Monomania, Mania, and Dementia; the first recently composed or at least revised, the second dated in 1818, and the third in 1814. Among other inconveniences hence resulting, the reader is disposed to conclude that M. Esquirol considers *folie*, hallucinations, illusions, fury, melancholia, mania, and monomania as so many separate disorders. This is particularly the case as regards *la Folie*, by which we understand insanity, and *la Manie*, by which we should understand the same, if the observations made under this head did not show that by the latter term M. Esquirol means a distinct affection. We conclude that la Folie is intended to express general insanity, and la Manie only its maniacal form; but the definitions given of these terms afford little help, being nearly word for word the same: the only important difference being that la Manie is described as a state marked by excitement.

"La folie, l'aliénation mentale, est une affection cérébrale, ordinairement chronique, sans fièvre, caractérisée par des désordres de la sensibilité, de l'intelligence, de la volonté.

"Je dis ordinairement, parce que la folie est quelquefois d'une courte durée, parce qu'au début et dans le cours de cette maladie, il se manifeste des symptômes fébriles." (Vol. i. p. 5.)

"La manie est une affection cérébrale, chronique, ordinairement sans fièvre, caractérisée par la perturbation et l'exaltation de la sensibilité, de l'intelligence, et de la volonté. Je dis ordinairement sans fièvre, parce qu'au début, quelquefois dans le cours de la manie, on observe des symptômes fébriles qui peuvent en imposer, et qui rendent difficile le diagnostic. (Vol. ii. p. 132.)

Whatever may be objected to their arrangement, the treatises are all full of facts of the most interesting description, gathered in the course of long experience and with unusual opportunities, detailed with clearness and force, and accompanied by reflections indicative of the acute and judicious character of the author's mind.

Dr. Prichard's treatise was among the first of those of which the origin has been attributed, by the writers, to the Cyclopædia of Practical Medicine; and if no more direct benefit should have been communicated to the profession by that comprehensive publication, (which we are, of course, very far from asserting,) the indirect advantages arising, and to arise, from the volumes which it has suggested, are and will be such as the editors must always contemplate with profound satisfaction. Dr. Prichard's rank among the most distinguished living medical authors is too well known to require any laboured introduction of him to the notice of our readers. The general character of his treatise may be said to be, that it contains the best information which is at present possessed on the various matters of which it treats. It is a fair, clear, and admirably condensed compendium of documents and statements from various authorities, collected in various establishments for the insane, in various countries; so placed together, and with so much discrimination commented upon, as to convince the reader that the first object of the author has been to elicit truths on which the practitioner, the moralist, the jurist, and the legislator might rely.

In his introductory chapter, after noticing the unsuccessful attempts at definition made by several previous writers, Dr. Prichard quotes with approbation the observation of Pinel, that insanity consists, in certain cases, in a morbid perversion of the affections and moral feelings exclusively, and without any perceptible lesion of the intellectual faculties; an observation which he considers of the highest importance, pathologically and practically, and which he takes much pains to confirm. Dr. Prichard's division of insanity, therefore, is, first, into *moral* insanity, and secondly, *intellectual* insanity; the latter being subdivided into *monomania*, or partial insanity; *mania*, or raving madness; and *incoherence*, or dementia.

This division is simple, and seems to admit of useful practical application: it also possesses, as expressed by Dr. Prichard, the great recommendation of conveying intelligible ideas, in English words. We concur in the opinion expressed by the author, and by Pinel, that there are cases of insanity in which the disorder is chiefly shown in a disturbance or a depravement of the feelings and affections; cases, indeed, which, in their slighter forms, present little but eccentricities of feeling or of temper, exercised, in different examples, in benevolent or in spiteful modes of action; but in many cases, without any general suspicion of insanity of mind; producing effects which destroy the happiness of families; the consequences of what are commonly spoken of as extreme or habitual bad temper, or capriciousness as regards the attachments of friendship or relationship. This kind of moral insanity begins in the slightest departures from healthy feeling, and may be traced through every variety of shade to forms of severity in which it is so evidently associated with an infirm, ill-judging, ill-reasoning, perverted mind, as to leave some doubts, in our estimation, whether the slightest forms of moral insanity are not in reality connected with some proportional defect in the mental powers. We must confess, indeed, that, great as is our deference to Dr. Prichard's superior observation, we have some difficulty in admitting the existence of any form of moral insanity disjoined from some degree of mental infirmity, less perceptible in the slighter cases, but manifest in the severer; in some instances merely marking out an individual, in others characterizing all the ramifications of a family, and in the latter case illustrating almost every possible variety of moral disorder and mental peculiarity. One is immoral, one is extravagant, one is morose, one is ever pursuing fancied discoveries; and none is of sound mind. The connexion of these cases with a certain mental character seems to us to be shown in this, that whatever palpably affects the mind,—as drinking wine or spirits, for instance,—brings out the more the peculiarity of the affection, making one more openly immoral, another more thoughtlessly extravagant, another more dangerously morose, another more wildly speculative: showing that the disordered affection shoots out the more in proportion as the controlling mental power is removed; and rendering it probable that its first springing up in the character was attributable, at least in part, to some want of mental balance:—we say in part, because we presume that a disposition to be more or less affected by particular impressions, moral as well as physical, may, or rather must, arise from the original constitution of the nervous system of the individual; of that part of the frame, whatever it be, on which such impressions are made, or in which the moral feelings may be said to reside. Even in the case adduced by Dr. Prichard, in which the insanity consists merely of violent gusts of anger, we should lean to the old belief, embodied in an ancient proverb, and attribute these sudden storms of feeling partly to the want of mental power to control them, and partly, of course, to an inherent morbid excitability. We cannot conquer, what may be a prejudice in us,—a belief that in all such cases the intellectual faculties are somewhat unsound. It is also worthy of remark, that *whenever* the mental faculties are disordered,—that is to say, in every case of insanity, the moral feelings and affections are to some extent impaired: their perversion, indeed, is often the first symptom of mental derangement, and ushers in every successive attack. In all the intervals in which a man is in sound mind, he may live happily with his wife, and be indulgent to his children: when the attack of insanity is coming on, he treats his wife with cruelty, and threatens the life of those children. M. Esquirol truly remarks, that a return to the usual feelings is the surest sign of a restored mind. The question is, whether or not the return of the feelings is the *effect* of the restored mind; whether the original fault is a proneness to excess in the feelings and affections, good or bad, or a defect in the mental power. There is, at all events, an evident disproportion in such cases between the feelings and the controlling faculties, and the practical importance of this class of cases is not less than that which Dr. Prichard attaches to them; and, if authority alone can decide the question, we must acknowledge the weight attached to that of Dr. Prichard and of Pinel. Dr. Prichard seems to think that M. Esquirol has also, after some hesitation, adopted the opinion of the latter, that there is a *manie sans délire*, a *folie raisonnante*, an insanity which exists not only without intellectual delusion, (that old and mischievous notion, not yet quite exploded,) but without intellectual error.

M. Esquirol's opinions are so scattered through the several memoirs of different dates, which form the principal part of his two recently published volumes, that we scarcely know where to look for his latest views on any single point: but, in the commencement of his second volume, when treating of monomania, in pages written since the publication of Dr. Prichard's work, to which he refers, he combats the notion of the intelligence being in a state of integrity in what Dr. Prichard calls moral insanity, although he maintains that there is a reasoning mania and a monomania. Our belief is, that the understanding is disturbed in all the cases, only more in some and less in others; on many subjects in one, in others on one subject; and, for the most part, for the very reasons which Esquirol adduces against the belief in a mere moral insanity. "If it were not so, the lunatics would let themselves be guided by reasoning, and would see that their principles are false and their actions unusual and eccentric. Their intelligence is more or less in fault; it has lost its influence over their will, it is no longer in harmony with the other faculties." (*Esquirol*, vol. ii. p. 5.)

The cases which seem to us, of all that are alluded to by Dr. Prichard, as most countenancing this view, are those in which the only departure from rational modes of action is shown in a fondness for every kind of unmeaning mischief; or for an equally unmeaning love of destroying everything within reach; or for appropriating articles which belong to others, without any probable motive save that of a disordered state of the moral feeling, as when theft is practised by persons of property, or when a patient will take no food unless what he considers to be stolen. Cases of inordinate penuriousness may perhaps be classed with these. Certain perversions of natural feeling, which subject the depraved individual to the treatment of a criminal, should, we think, always be considered to belong to insanity. Dr. Prichard alludes to them as constituting cases of moral insanity: they may perhaps belong to disordered sensation. They are generally associated with some eccentricity of character, but, as has been shown by too many instances, may arise in minds capable of a high degree of intellectual acquirement; and yet which, in certain circumstances, resign the actions of the individual to the guidance of his disordered propensities alone. The *delirium senile*, or insanity of old people, is surely very generally accompanied by a weakened intellect.

It is important to remember that, in these cases of moral insanity, the prognosis is generally unfavorable, unless arising from any external and accidental cause, which admits of removal, or from the influence of which the patient can be abstracted or defended. In other cases "the disease is likely either to be permanent or to terminate in another form of insanity."

Several cases are related by Dr. Prichard to illustrate the description of moral insanity and monomania, the relation between them, and the transition from one to the other. A careful perusal of these cases has failed to set aside our doubts respecting the alleged existence of moral insanity separate from some disorder of the intellect. In most of the cases there were, as it appears to us, many indications of an ill-judging and weakened mind, and in most of them intellectual disorder of a more marked character supervened. The highly respectable testimony of Dr. Wake of York, of Dr. Bompas, Dr. Fox, and Dr. Symonds, of Bristol, and of Mr. Hitch, of the Gloucester Lunatic Asylum, is adduced in support of the view taken by Dr. Prichard. Mr. Hitch says that such cases have long been recognized in the asylum, and the individuals so affected have been termed there "insane in conduct, and not in ideas." The question seems to turn upon the degree in which the habitual conduct of all individuals depends upon mental determinations. We ourselves incline to the belief that our feelings and affections are the joint result of certain emotions which have been made the subject of intellectual actions, or of reflection; and, when these feelings or affections become perverted or depraved, we can hardly in any case suppose that the mental actions continue to be correctly performed. Such, it appears to us, was evidently the case in the first of Mr. Hitch's interesting illustrations: it is said of the patient, that "on business he would converse most rationally; but, if the opportunity had presented itself, would have expended his money in the most useless purchases. He was capable of making the nicest calculations connected with his own affairs, and was correct in all his data when speaking to a second person; but, when left to himself, his conduct and language were ridiculous in the extreme." Such also appears to us to have been the case in the little girl whose condition is so admirably described in the same communication: a child of seven years of age, who, from being mild, affectionate, and clever, became "rude, abrupt, vulgar, and perfectly unmanageable," despised her parents, was cruel to her younger sisters, would eat her own fæces, drink her own urine, and "swear like a fishwoman." This child, too, was of a family in some of the branches of which insanity had existed; and her bodily health was much disordered. "I could never detect in her," says Mr. Finch, "any fixed idea, either of fear or belief, which influenced her conduct. She acted from the impulse of her feelings, and these were unnatural and unhealthy:" uncontrolled, we should add, by the faculties of her mind, by the recollections of what she had been taught was decent and proper, and by her judgment. She knew and avowed that her actions were foolish and disgusting, but could not refrain from them. Surely, then, the controlling power was weakened; and the only question is, whether or not that controlling power resides in the intellect?

The sections in which Dr. Prichard describes mania, or raving madness; the phenomena of protracted insanity, including that excited form to which paralysis so generally succeeds,—the subject of an excellent treatise published by M. Calmeil in 1826, (De la Paralysie considérée chez les Aliénés;) and incoherence, or dementia, are examples of condensed and graphic delineation, in which everything is well arranged. There is no unnecessary dwelling on insignificant points, no useless repetition, and the impression left on the mind is clearer than what is commonly derived from writings on such subjects; the diversities of which seem to defy the powers of arrangement possessed by common authors. In the section on Incoherence, the distinction, often neglected by medical writers, of the incoherence consequent on age or disease, from idiotism, or natural imbecility of mind, is well pointed out. In one case the faculties are lost; in the other they never existed: in one case broken recollections of the past remain; in the other there are no recollections, and the mind is as that of an infant. The causes of incoherence, or dementia, when not congenital, are illustrated by a table exhibiting in the first column the number of such cases admitted into the Salpêtrière, in 1811 and 1812; and the second those admitted into M. Esquirol's private establishment.

TABLE OF CAUSES.

| *Physical Causes.* | *Number of* | *Individuals.* |
|---|---|---|
| Disorders connected with the catamenia | 11 | 4 |
| Critical period | 29 | 6 |
| Consequences of childbirth | 5 | 3 |
| Blows upon the head | 3 | 0 |
| Progress of age | 46 | 3 |
| Ataxic fever | 1 | 2 |
| Suppression of hemorrhoids | 0 | 2 |
| Mania | 14 | 4 |
| Melancholia | 13 | 2 |
| Paralysis | 3 | 2 |
| Apoplexy | 3 | 2 |
| Syphilis and abuse of mercury | 6 | 8 |
| Faults of regimen | 0 | 6 |
| Intemperance | 9 | 6 |
| Masturbation | 4 | 7 |
| *Moral Causes.* | | |
| Disappointed love | 1 | 4 |
| Fright | 4 | 3 |
| Political excitement | 0 | 8 |
| Disappointed ambition | 0 | 3 |
| Poverty | 5 | 0 |
| Domestic griefs | 8 | 4 |

Dr. Prichard divides incoherence into four degrees: the first is that of *forgetfulness*, or loss of memory, so commonly exemplified in old persons with regard to recent events; and sometimes occurring in individuals at an earlier age, from various sudden impressions to which they have been subjected. The second he terms the stage of *irrationality*, or loss of reason; a state which is generally long continued, the physical health remaining good, but from which there are some rare cases of recovery, attended with circumstances deserving the attention of the practitioner.

"Pinel informs us that many, especially young persons, who had remained in the Bicêtre several years or months in a state of absolute fatuity, have been attacked by a paroxysm of acute mania, of twenty, or five and twenty, or thirty days' continuance. 'Such paroxysms,' he adds, 'apparently from a reaction of the system, are in many cases succeeded by perfect rationality.' The same result has been observed on the restoration of demented persons, or of maniacs in the advanced stage of insanity, after severe attacks of fever attended with delirium. Such attacks are often fatal to lunatics; but, of those who recover them, not a few are subsequently restored to the possession of their faculties." (p. 96.)

The third degree of incoherence is that in which the individual is incapable of comprehending anything that is said to him. Dr. Prichard says it may be styled the stage of *incomprehension:*

"Physical activity is often remarkably displayed by persons reduced to this state, and it assumes the appearance of trick or habit. Some jump or run to and fro, or walk round perpetually in a circle. Some dance or sing, or vociferate frequently. Many talk incessantly in the most unmeaning jargon, attaching no ideas whatever to their words; others pass their time unceasingly in muttering half sentences and broken expressions, in which it is scarcely possible to discover any link of connexion; or, if any association can be traced in their thoughts, it is of the most trivial kind, and depending on a word or on some sensible object which for a moment attracts a degree of attention. Many, on the other hand, sit in silence, with a sedate and tranquil look, sometimes with a vacant smile or with an unmeaning stare, and scarcely pronounce a syllable for weeks, or months, or even for years. A few remain crouched in a particular posture, which they always prefer, though to bystanders it seems the most uneasy, and even painful: if placed in a different manner by those who have the care of them, they soon resume their habitual position. Many demented persons crowd round a stranger who happens to visit a lunatic asylum, and gaze at him, having just enough of intelligence to perceive something new and to which they are not accustomed in his aspect. Some individuals in this state have a propensity to adorn themselves in a strange manner: they take anything that happens to be in their way, and append it to their dress, which is singular and ridiculous.

"M. Esquirol has described in a striking and accurate manner the aspect of countenance peculiar to dementia, and especially belonging to this stage of the disease. 'To the disordered state of the intellectual faculties,' he says, 'the following symptoms are added: the face is generally pale, the eyes moistened with tears, the pupils dilated, the look wandering, the countenance motionless and devoid of expression; frequently the muscles of one side are relaxed, and give the face a distorted appearance; the body is sometimes lean and emaciated, at others it is loaded with fat: in such instances the face is full, ruddy, the neck short. In a few persons no outward sign can be perceived indicative of the decay of intelligence." (p. 97.)

The fourth degree of incoherence is that of *inappetency,* or loss of instinct and volition; a state in which even the animal instincts are lost, and the individual has merely organic or physical existence; appearing scarcely conscious of life, having neither desires or aversions, and being unable to obey the calls of nature. Whoever has had opportunities of observing lunatics must recognize the fidelity of the following sketch of a group of persons reduced to this miserable condition.

"Scarcely any exhibition of human suffering can be more deeply affecting than the aspect of a group of lunatics reduced to the last stages of fatuity, and those who have never witnessed such a spectacle can hardly imagine so abject a state of mental degradation. In a group of this description, an individual may be seen always standing erect and immoveable, with his head and neck bent almost at right angles to his trunk, his eyes fixed upon the ground, never turning them round, or appearing by any movement or gesture to be conscious of external impressions or even of his own existence. Another sits on a rocking chair, which she agitates to and fro, and throws her limbs into the most uncouth positions, at the same time chanting or yelling a dissonant song, only expressing inanity of ideas and feelings. Many sit constantly still, with their chins resting on their breasts, their eyes and mouth half open, unconscious of hunger or thirst, and almost destitute of the feelings which belong to merely physical life; they would never lie down or rise were they not placed in bed and again raised by their attendants. A great proportion of the patients who are reduced to this degree of fatuity are found to have lost the use of their limbs in a greater or less degree by partial or general paralysis.

"From such a state it is scarcely imaginable that recovery ever took place, but patients in the last stage of fatuity often linger for many years. Their state, however, is not always uniform: some of them have comparatively lucid intervals, in which nature seems to make an effort to light up the mind and recall lost impressions and ideas. I have often observed a patient who sits all day in a wooden elbowed chair, with his chin hanging over his breast, appearing hardly conscious of existence, and unable to assist himself in the calls of nature, who would not eat if food were not actually put into his mouth. He has been for several years in the same state, except that he occasionally appears to rouse himself, and for a short time to recover an unusual degree of animation. At such periods he will sometimes read a chapter in the Bible with a clear voice and a distinct and intelligible articulation. Such occasional variations in the state of demented persons are not infrequent. They are capable of being raised by favorable influences from a lower degree of their disease into one which is above it in the scale." (p. 98.)

M. Esquirol is at some pains to combat the opinion advanced by M. Foville, and maintained by others, that there is no such thing as a case of real monomania. Cases to which such a denomination could be correctly applied are, we think, very rare; for, although the unsound state of the mind may, and is in many instances, chiefly manifested in relation to some particular illusion or belief, careful investigation would seldom fail to discover coexisting proofs of an impaired understanding. Dr. Prichard is of opinion that the monomania commonly supervenes upon some degree of moral insanity; and gives cases in illustration of this opinion. We cannot see any good reason for distinguishing the case of a person who thinks she can regulate, like the philosopher in Rasselas, the course of the sun (Esquirol, vol. ii. p. 8), nor those of individuals who think themselves emperors or princes, from cases of general mania. A patient who considers himself in audible communication with God and with angels can scarcely be considered as only mad on one subject: his sense of hearing is subject to illusions, and his mind to hallucinations, (to adopt M. Esquirol's terms as expressive of sensorial deceptions and mental reveries, although the difference here again is more refined than real:) if he writes, he hears voices dictating to him; he neglects all the ordinary customs of religion; and when he speaks, he believes himself the mere echo of a voice proceeding from an angel. Such an individual may be capable of writing prose and verse correctly, but there must be so much disorder in his perceptive and reasoning faculties as scarcely to make it possible that he should be found rational in all parts of his conduct unconnected with these striking delusions. In other cases related under this head by M. Esquirol, we can only see examples of general mania and melancholia; upon which some particular delusion supervened. A merchant, long remarkable for his wayward and suspicious temper, rash and unfortunate in his speculations, disordered in his health, and hypochondriacal, always apprehensive of poison lurking in his food, quarrelsome with an indulgent father, jealous of an affectionate wife, reads an account of a pretended dauphin, and immediately imagines himself to be the real dauphin, and forces his way into the Tuileries, to claim his rights. Although he persists in this assertion, makes proclamations to the people, and writes a plausible history of his pretended birth, we can only regard all this as a new feature supervening in a case of insanity; an instance of delusions displacing other delusions, and not to be called a monomania, without a confusion of terms.

Persons of conscientious and timid character often experience an excessive apprehension of being considered capable of acts of unfairness or dishonesty, and particularly in shops or warehouses in which they are placed in the midst of much moveable wealth. The strongest instance of these feelings being carried to excess, and at the same time one of the least questionable cases of monomania, is related in this section of M. Esquirol's work. An unmarried lady, thirty-four years of age, of lively and mild disposition, sanguine temperament, with blue eyes, auburn hair, and florid complexion, had been brought up, as is often the case on the continent, in commerce, and had always been scrupulously afraid of taking any advantage of others, in matters of business, and in making out accounts. It was her frequent custom to pay a visit to an aunt, without her bonnet, and dressed in an apron which she usually wore. When about eighteen years of age, she evinced an extraordinary uneasiness, when coming away from her aunt's house one day, respecting the possibility of bringing away anything belonging to her aunt in the pocket of her apron; and after that time, she always paid her aunt visits without her apron. She became more and more afraid of making out unfair accounts; and when touching money, of retaining any part of it between her fingers. She acknowledged that this scrupulous fear was ridiculous, but said that she could not help feeling it. At length she was obliged to give up her commercial occupations. Her disquietude and her fears became more general. After touching anything, she washed her hands in a great quantity of water. Her constant care was to touch nothing with her hands or her clothes; and she contracted the custom of rubbing the fingers of each hand against the other, as if to free them from something under the nails. She carefully refrained from opening doors, windows, and closets, because something of value might be attached to the handles or the keys, and adhere to her hands. She carefully examined every chair or seat, before sitting down; wore the tightest shoes that she could force her feet into, in order that they might contain nothing concealed. After many amendments and relapses, this lady has now been for some time under the care of M. Esquirol. During eighteen months she appeared tolerably free from the peculiarities above mentioned, and the action of rubbing the fingers was seldom observed; but since June, 1837, says M. Esquirol, writing this account of her six months later, the phenomena have appeared with greater intensity, and this daily increases. In order to give a more lively idea of her state, he describes her habits during one day. She always rises at six; and an hour and a half, and sometimes three hours are passed at the toilette. Before getting out of bed, she rubs her feet for ten minutes, to remove anything that may have got between the toes or under the nails; she then carefully examines her slippers, and desires her maid to do the same, to ascertain that they contain no valuables. Her comb is passed many times through her hair, for the same purpose. Every article of dress, and every fold, is cautiously inspected, with numerous rubbings of hands at intervals during such inspection; and all this done with fatigue and extreme anxiety; and if omitted, the omission leaves her ill at ease all the day. At ten she is ready for breakfast, when the napkins, plates, glasses, jugs, and knives undergo the same kind of scrutiny. At dinner, the same precautions are taken. Her preparations for going to bed are as minute as those of the morning toilette. During the day she reads, or works with her needle; but these and all other occupations in which her hands are brought in contact with any other object, lead to renewed rubbings of the fingers. If she writes, the paper is shaken, and the pens, and the writing-desk, and she never seals a letter without being assured by her attendant that there is nothing folded up in it. If alone in a room, she will not sit down until some one comes and assures her that there is nothing on the chair to prevent her so doing. She pays and receives visits, but always contrives to practise her cautions; and when she goes to see her relatives in her native town, she so arranges her journey as to arrive early in the morning, to enable her to change her dress completely before seeing her friends. With all this, she never talks unreasonably, but is aware of her peculiarities; she even sees the absurdity of her scruples and fears, and laughs at them herself; or sometimes she groans and weeps over them. She tries to overcome them, and even points out the means, often such as are very disagreeable to her, of getting the better of them. She goes to shops with her servant, never touching money herself: she goes to the theatre and the public promenades; makes parties to go into the country, and joins a social circle every evening. Her conversation is lively, clever, and sometimes sarcastic (*malicieuse*); but if she changes her chair, or touches her head, or her dress, or if any one comes into or goes out of the room she rubs her fingers diligently. Her health is good. It is impossible, M. Esquirol says, at any time, to detect the least disorder of the sensations, in the reasoning, or in the affections of this interesting patient.

We have mentioned the particulars of this case, as illustrative of pure monomania: extreme conscientiousness appears to furnish its moral peculiarity; but we should hesitate to pronounce it, with M. Esquirol, unmarked by a morbid state of the senses and of the understanding or reason: and we cannot recognize the justness of the term *manie raisonnante* (reasoning mania) applied to cases of this kind by Pinel, or the term moral insanity, given to them by Dr. Prichard. In this instance the moral peculiarity was the probable origin of the malady; but surely impaired sensorial functions and reason became complicated with the morbid conscientiousness. It is no less unquestionably incorrect, we conceive, to call any kind of mania a reasoning mania: the reason in this case, for instance, is correct on ordinary matters; it even retains power enough to point out the absurdity of the patient's conduct; but it is not powerful enough to prevent that conduct: to that extent, therefore, it is impaired. It is overmastered by an undue activity, apparently, in one portion of the brain. The reason of the lunatic in "reasoning monomania," says M. Esquirol, is not essentially injured, for it comes in aid of the lunatic's actions, and the patient is always ready to justify his deeds and sentiments." These circumstances, it appears to us, warrant exactly opposite conclusions.

M. Esquirol and Dr. Prichard are zealous anti-phrenologists; but we incline to think that the phrenologists could give a better account of some of these cases than they. But upon that debateable ground we have no wish to enter; seeing that it is too much occupied with prejudices, political and religious, pitiless and fierce, to allow the combatants room and fair-play. It touches besides, too dangerously upon too many of those subjects upon which mankind rejoice in conventional delusions; it lays bare too much the sophistry which shrouds many artificial social relations whereupon men fear

> " Lest their own judgments should become too bright,
> " And their free thoughts be crimes, and earth have too much light."

The monomania, if it can properly so be called, of drunken persons, of incendiaries, and of homicides, is forcibly depicted by M. Esquirol: of these varieties, the examples are numerous; and they touch on the delicate boundary where the extent of moral responsibility is difficult to be defined, and the right of human punishments controvertible. The exact limits of these lamentable cases require to be pointed out by bold and enlightened moralists; and the inconveniences resulting from them to be dealt with by humane and courageous legislators. Publicly putting half-lunatic criminals to death has, we apprehend, done little to promote the security of mankind, and still less to improve the humanity of the spectators of such dreadful inflictions. In some future age, the prevention of crimes will perhaps occupy more attention than their punishment.

A section of Dr. Prichard's treatise is devoted to the consideration of General Paralysis complicated with insanity, first pointed out to physicians by M. Esquirol in the Dictionnaire des Sciences Médicales; a form of paralysis more frequent in men than in women, and more frequent among patients of the cultivated classes than among the poor. At Charenton, the proportion of paralytics is one sixth of the whole number of admissions. We had a few years ago an opportunity, with the assistance of M. Calmeil himself, of observing in that admirable asylum, the various forms or degrees of this disorder in lunatics, of which his work contains such a faithful description. In the first degree of the malady, its nature is indicated chiefly by an impediment in the movements of the tongue, a slowness and difficulty in articulating syllables; but the tongue can be steadily protruded, and the muscles of the face are not distorted. In the second degree, all the voluntary muscles, and the sensations, are affected: the patients rise up slowly, and walk with difficulty; and slight impressions upon the skin and nostrils are disregarded. In this stage, the functions of physical life are nearly in a healthy state, but the patient's condition is dirty and miserable. In the last deplorable degree of this complication, " these patients, motionless and insensible, are reduced to a state of mere vegetation; their existence is a kind of slow death." It is remarkable, however, that the death of these patients is seldom, as M. Calmeil observes, the simple consequence of the cerebral disease; but is occasioned by the formation and suppuration of pulmonary tubercles, and by inflammation and ulceration of the intestinal canal. The paralysis itself is not, in these cases, the result of effusion of blood in the brain, of acute ramollissement, or of sanguineous congestion, but almost uniformly of chronic inflammation of the substance of the brain, especially at its surface; sometimes complicated with congestion in the brain, hemorrhage in the cerebral substance, or between the two laminæ of the arachnoid, ramollissement in some part of the brain, or erosions of the cerebral surface. The mean duration of such cases is a little more than twelve months. The complication, which is certainly of common occurrence in Paris, appears to be more rare in the south of France, and seldom to be seen in the great asylum at Aversa in Italy. It has not been much observed in this country; but Dr. Prichard says that the result of several enquiries made by him on the subject is an opinion that it is by no means uncommon in our public institutions, although comparatively rare in private asylums. M. Esquirol relates the case of a patient who was brought to him, with the hope that he might give a promise of his recovery; but in which he gave a most decidedly unfavorable prognosis at the first interview; chiefly founded on the observation of a slight degree of difficulty of articulation with which the mental excitement was complicated. The rapid progress of these cases is, he observes, very striking.

The symptoms and character of Mania are described with great force and fidelity in M. Esquirol's second volume.

"The face of maniacs is flushed and swelled, or it is pale and contracted; the hair is set on end (hérissé), the eyes are brilliant and haggard: the patients shun the light and have a horror of certain colours; they have noises and ringing in their ears; their ears are sometimes very red: the slightest noise excites them. Monomaniacs have headach, heat in the interior of the cranium, anorexia, or a voracious appetite. Consumed by internal heat, they are tormented by a devouring thirst for cold drinks; they have a burning sensation in the bowels, constipation, insomnia, or, if they sleep, frightful dreams and sudden awakings.

"Maniacs are remarkable for false sensations, illusions and hallucinations, a faulty association of ideas, which are rapidly reproduced without order or connexion; they are remarkable for the errors of their judgment, the perturbation of their affections, and the violence of their will. They have great nervous excitement; their delirium is general, all the faculties of the understanding are excited and overturned, (*exaltées et bouleversées*); and every thing that excites an impression, either physical or moral, upon them, including the vain products of their imagination, excites them and becomes the subject of delirium." (Vol. ii. p. 133.)

The diverse modes in which the patients express their ideas in maniacal affections, and their various and expressive gestures are well described by M. Esquirol, (vol. ii. p. 151); and yet no description can comprehend all the singular varieties observed among them. Some repeat one word again and again for many hours; some speak of themselves always in the third person; and we have known some hold long and objurgatory colloquies, in two feigned voices. Some invent a peculiar language; some of course vociferate and sing. All these peculiar modes of speech are but so many expressions of shades of mental excitement or oppression; readily comprehended by persons of strong feeling, albeit not mad. The remarkable alterations of the countenance, which have attracted less observation, are graphically described by Esquirol; and some of them are terrifically represented in the plates.

The erroneous notion that all lunatics can bear cold and hunger with impunity was long since refuted by M. Esquirol. Some of the patients, apparently tormented with feverish heat, delight in exposing themselves freely to cold air or cold water; but many of them suffer much in the winter, and would die if not guarded from severe exposure. A disordered state of the digestive organs often renders them averse to taking food; and on this point M. Esquirol makes an observation of some importance; namely, that he has never seen any bad consequences arise from the refusal to take food in *mania*, although, in *monomaniacs* and in the *melancholic*, hunger is resisted with a hurtful and even fatal stubbornness. Want of sleep, and constipation, and sometimes, with worse effects, diarrhœa, are common symptoms in the maniacal.

Long experience has taught M. Esquirol, that amidst all the violence and apparent irregularity of mania, it observes, for the most part, a regular course; and this he has endeavoured to describe. We think practitioners in general are little acquainted with this part of the subject. Maniacal patients are commonly removed to asylums as soon as they become troublesome, and, except to those accustomed to the care of them, nothing is more perplexing than to be called upon to say what the chances of recovery are in any case, or how long the disorder will continue, or what will be its character in its progress. These are points on which it is well worth while to attend to the opinions of M. Esquirol.

"Mania," he says, "has its precursory symptoms: we distinguish three periods. In the first, the patients complain of general and undefinable uneasiness, headach, heat of the cranium, burning feeling in the entrails, pain of the epigastrium, disgust for food, thirst, constipation: they have internal agitations, vague inquietudes, insomnia, reveries, presentiments, alternations of gaiety and sadness, and sometimes a transient delirium; but they yet retain affection for their parents and friends. The symptoms increase; the delirium becomes general and permanent, the moral affections become perverted. The passage to this second period is marked by some acts of violence or of fury, spontaneous or provoked: after a time, most frequently after a considerable time, the maniac becomes calm, less turbulent, less disposed to fury; he is more attentive to outward impressions, and more docile when advised. At length the moral affections reawake, the traits of the countenance become less agitated (*convulsifs*), the emaciation diminishes, the patient sleeps longer, and is conscious of his state. Ordinarily, in proportion to the reestablishment of the functions of nutritive and relative life, a more or less complete crisis takes place; but if the functions of nutritive life reestablish themselves, and the delirium does not proportionably diminish, we have reason to fear that the mania will pass into the chronic state, and degenerate into dementia." (Vol. ii. p. 158.)

The progress of mania is, however, not always quite so regular.

"We have seen," continues Esquirol, "that this disease varies in its mode of invasion. It varies in the succession of the symptoms, in their duration, in their termination. Sometimes the mania attains its highest period from the very beginning, and continues to the end of the attack, which takes place all at once. The patient seems then like a person who has come out of a dream; it appears to him as if some obstacle which isolated him from the external world has been torn, or has fallen from before his eyes. Sometimes, a progressive diminution of the number and intensity of the symptoms foretels the approaching solution of the malady; sometimes the patient only arrives at convalescence by a succession of remissions, of greater or less completeness and duration. One point to which I cannot too strongly call attention, is the remission which is observed in the course of the first month from the invasion of the mania: this remission is constant. Does it mark the cessation of the period of irritation?" (Vol. ii. p. 167.)

Mania shows itself most in persons of robust and plethoric constitution, of the sanguine or nervous temperament. The individuals affected are often possessed of great susceptibility, a lively and irritable character, disposed to anger, with an ardent imagination; embracing extravagant projects with enthusiasm, and giving themselves up to hazardous speculations. Some of the patients are found to have been subject to hemorrhages, cephalalgia, dreams, somnambulism, nervous affections, hysteria, convulsions, epilepsy, and cutaneous diseases.

The mania of epileptics has a peculiar character: it comes on after the paroxysms, and lasts either a few hours or a few days. Of four hundred epileptic patients at the Salpêtrière, fifty are maniacal after the paroxysm. Their fury is more dangerous and more to be feared than that of any other lunatics.

Mania is sometimes preceded by melancholia or hypochondriasis. Sadness, distrust, general uneasiness, pains of the limbs or head, and an apprehension of some serious illness, or even of madness, are sometimes the precursors of mania.

Mania does not often suddenly break out; but yet it is the most frequent form of a sudden attack. In general, some irregularity in the affections is first perceived; and these, we doubt not, are preceded by some disordered mental movements, which only become manifest in these results. By slow degrees, and through every kind of irregular conduct, the disorder arrives at its height. Sometimes a violent attack of mania is preceded by a state of stupor and helplessness. The patients are motionless, remain where they are placed, require to be dressed and fed; their eyes are brilliant and the face contracted. Then the mania suddenly and violently declares itself. In other cases, after the disappearance of some habitual indisposition, the individual feels a perfect sense of well-being, thinks himself to have attained the most perfect state of health; feels possessed of an extraordinary degree of strength and of happiness: all nature appears more beautiful in his eyes; and everything seems possible. Insomnia, constipation, agitation, progressively ensue; the ideas become confused, and "the patient enters gaily into the most frightful of maladies." (*Esquirol*, vol. ii. p. 146.)

As respects the actual state of the mental faculties in mania, M. Esquirol considers that it chiefly consists in a defective power of attention; or, as he expresses it, in a defect of harmony between attention and the actual sensations, ideas, and recollections. (Vol. ii. p. 148.) This, we would beg to observe, is a defect subversive of accurate comparison; but a defective state of other faculties, as of the memory, or the imagination, may produce the same result.

There is nothing particularly satisfactory to us in the short section introduced by Dr. Prichard on the state of the sensorial and intellectual functions in such cases. Dr. Prichard reverts to Pinel's opinion of the primary disorder, in many instances, of the moral constitution, and quotes the passages in which Broussais has illustrated the devouring character of theory by ascribing this moral perversion to an irritation of the trisplanchnic apparatus, and especially in that of the stomach, acting on the brain. Guislain's endeavours to determine the particular intellectual process which undergoes the modification characteristic of madness are mentioned with approbation, although it is pronounced to have failed,

"perhaps owing to the inscrutable nature of the research." Hoffbauer is cited as maintaining that many disorders of the intellect originate from certain defects in the power of attention; which is too concentrated in monomania, and altogether distracted in mania. This observation is undoubtedly true of many cases; but we presume that the cause of the impaired attention is sometimes an over-excited imagination, sometimes a prepossession of the mind by some morbid train of ideas by which the attention is enchained, sometimes a delusion of the sense, and sometimes an impairment of the memory; the constant effect still being this defective attention noticed by Hoffbauer, and by others; a state incompatible with just comparison, and consequently with the exercise of judgment.

The connexion of any particular state of the physical functions with insanity has not been more satisfactorily established than the precise condition of the sensorial and intellectual functions. A disordered state of the alimentary canal is the most common; but the nature of the relation of this to the mental disorder is not established. Acute cases are commonly attended with febrile symptoms, and excitement of the heart and arteries, and with sleeplessness. Other cases supervene gradually, with a torpid state of the natural and vital functions. Customary discharges. and external and internal affections, sometimes disappear when insanity comes on, and reappear or resume their progress when the mental disorder subsides:

"The catamenia, if not suppressed previously to the manifestation of maniacal symptoms, soon become scanty, or cease entirely after its actual appearance. Lochiæ and other analogous effluxes are suppressed ; ulcers, which had become habitual and had long discharged, are dried up ; chronic eruptions generally disappear, or are materially lessened; symptoms of pulmonary phthisis in various stages cease or become mitigated in a remarkable degree. On the decline of mental disorders, it is often found that the return of such discharges, or the revival of suspended trains of morbid phenomena, is the harbinger of restoration to a sound state of mind, though not to complete bodily health." (*Prichard*, p. 125.)

All who have been in the habit of visiting lunatic asylums must have become familiar with the faces of many of the inmates, who have been in confinement ten, twenty, or perhaps thirty years; and if we suppose that proper means have been adopted to promote their recovery, the view of the curability of such disorders must become extremely limited. Many of the cases, no doubt, have been from their very nature incurable, dependent on a peculiar constitution of the brain or on irremediable lesions. In some, it is not uncharitable to suppose that medical means have been too soon abandoned.

If recoveries take place, they generally take place early. Death not unfrequently follows not long after the invasion of madness. If neither of these events takes place, the patient sinks into a state affording very little hope, and generally set down as hopeless. The complication of insanity with other diseases materially affects the prognosis; and nothing makes it more unfavorable than the smallest indication of the general paralysis already spoken of. When the mental disorder has been preceded by attacks of epilepsy, the chance of recovery is not great. Mania is a more curable form than monomania or dementia. The number of cured at Charenton, out of 487 cases, was upwards of two fifths. All the reports and tables of lunatic asylums seem to establish the fact that recovery is much more probable in the early than in the advanced periods of the malady. The importance of proper treatment at such an early period, and the impropriety of sending the patient hurriedly off to a house of confinement, with no assurance of a proper system of treatment being persevered in, is very evident; but has been far too much overlooked. Nor are there wanting cases to prove that even after many years passed in a state of insanity the mind may recover; and that such recoveries have sometimes been overlooked or even concealed we have too much reason to suspect. Cases are related by M. Baumes and by M. Esquirol in which recovery suddenly took place after more than twenty years' duration of madness. Mr. Hitch, of Gloucester, to whom Dr. Prichard expresses himself indebted for so many particulars, has furnished him with tables showing the time that each case, of many cases, required for its treatment to effect recovery, and the length of time that the disorder had existed in each case prior to admission. The results are highly interesting. They show not only that the greater number of recoveries take place in the recent cases, but that in some, recovery took place in a short time after admission into the asylum, although the disease had been of long anterior duration. Of three male patients who had been insane ten years, one was cured in ten months, and the other two in six months; and one who had been insane forty years was cured in four months. Of two female patients who had been insane ten years, one was cured in nine months and the other in a year; and three other cases are recorded of cure after eleven, seventeen, and twenty years.

Generally speaking, the younger the patient is, the greater is the chance of recovery: and from the forty-fifth year to the end of life the number of recoveries progressively diminishes. Yet in the tables of Charenton, cited by Dr. Prichard, twenty men are marked as having recovered after the fiftieth year.

Mr. Farr introduces into his very able and searching pamphlet two Tables of Recoveries, one showing the total admissions and reported cures in twenty-six lunatic asylums, both English and French, the other showing the same circumstances in Irish District asylums, and at Haslar and Bethlem Hospitals. The proportion of cures in the highest of these (the Retreat at York,) is 50 per cent.; and in the lowest, (Hanwell,) 18. Exclusive of the Retreat, the highest proportion of recoveries is 46.2 per cent.; and the lowest, exclusive of Hanwell, is 33. Sir William Ellis ascribes the small number of recoveries at Hanwell to the chronic nature of the majority of the cases. We must refer the reader who is interested in the statistics of insanity to Mr. Farr's work, which is full of important facts.

Dr. Prichard has taken great pains to ascertain the proportional number of recoveries in general, and has inserted several tables illustrative of this point, although varying considerably from each other. Dr. Burrows has reported from his own experience a proportion of eighty-one cases of recovery in one hundred, and in recent cases of ninety-one in one hundred. Dr. Jacobi, of Siegburg in Westphalia, reports forty cures in one hundred cases, and six as alleviated. In the French asylums the proportion of recoveries is about one third of all the cases, and the same proportion seems to take place in the English asylums: but M. Esquirol says that the cures are more numerous in France than in England, (vol. i.

p. 93,) and he reproves his countrymen for supposing everything is best out of their own country; a fault of which they are not, we think, generally supposed to be guilty. Of late years, however, the proportion of recoveries at Bethlem Hospital has been considerably more than one half; but it is to be remembered that no cases are admitted in which the disease has already lasted longer than twelve months, and that the paralytic, the idiotic, the aged, and the infirm are excluded, as well as those discharged uncured from other hospitals. With such restrictions, the generality of the cases must be favorable to recovery; and Dr. Prichard very justly observes that the proportion of cures would probably be still greater, but for the regulation by which those uncured at the end of a year are discharged. The number of patients cured at the Salpêtrière in the *second* year is nearly as five in six to those who recover in the first year. "We are not," says Dr. Prichard, with his usual absence of exaggeration, "authorized to draw any positive conclusion on this subject, but we can hardly fail to entertain a strong doubt as to the propriety of the regulation still subsisting at Bethlem, and a suspicion that it is a relic of the ignorance of our predecessors which calls loudly for such a revision and adaptation to the improved state of knowledge, as would ere now have been obtained under our continental neighbours."

But the most encouraging table is that furnished by Mr. Tuke, of the Retreat near York; a table exhibiting the numbers of admissions into that asylum from 1812 to 1833 inclusive, with the results of all the cases. Of this table Dr. Prichard gives the following summary; first observing that the admissions of each year are divided into three classes, one for cases of less than three months in duration, a second for cases of more than three but less than twelve months, and a third for cases of more than twelve months: the fourth class contains cases of relapse re-admitted.

| Cases classed as above. | Recovered. | Died. | Removed. | Removed Improved. | Remain. |
|---|---|---|---|---|---|
| First class ............ 63 | 51 | 8 | 1 | 2 | 1 |
| Second class ......... 65 | 28 | 10 | 6 | 3 | 18 |
| Third class ...........101 | 31 | 15 | 17 | 4 | 34 |
| Fourth class .........105 | 58 | 17 | 13 | 1 | 16 |
| Total.............334 | 168 | 50 | 37 | 10 | 69 |

By this table it is seen that Mr. Tuke is warranted in estimating the recoveries in recent cases as nearly 7 in 8; but as it appears that some of those enumerated in the first class died soon after admission, of other disorders, and as others in the same class had in reality long been eccentric, and were consequently old cases, Mr. Tuke considers that the probability of recovery from insanity in recent cases is even greater than as nine to one. Our readers will do well to refer to the statements contained in this section of Dr. Prichard's work; for we believe the general opinion entertained with respect to cases of insanity, even of a recent date, is more unfavorable than ascertained facts permit us to retain; and the desponding view taken of such cases has an evident tendency to weaken the efforts of practitioners to get the better of this class of maladies. M. Esquirol (vol. ii. p. 177,) considers the maniacal form of insanity as offering the greatest chances of cure. The first attack is generally curable, if uncomplicated with epilepsy or paralysis. The second attack is also often cured; but the disease becomes infinitely more doubtful after the fourth. Of 269 maniacal cases cured, 132 were first attacks, 77 second attacks, 32 third attacks, and ten were cases in which the attacks had exceeded three in number. The duration of this form of insanity is also shorter than that of others: the greater number are cured in the first year, as is seen by the following Table of cures.

| | |
|---|---|
| Cured in the first month,.... | 27 |
| .... second........ | 32 |
| .... third ........ | 18 |
| .... fourth ........ | 30 |
| .... fifth.......... | 24 |
| .... sixth ........ | 20 |
| .... seventh ...... | 20 |
| .... eighth........ | 19 |
| .... ninth ........ | 12 |
| .... tenth ........ | 13 |
| .... twelfth ...... | 23 |
| .. in the second year.... | 18 |
| .... subsequent years | 13 |
| Total...... | 269 |

We would also strongly recommend to careful perusal the sections in Dr. Prichard's Treatise relating to the modes of death in lunatics, and to relapses and recurrent insanity. As in most other parts of the work, the author has in these sections condensed much practical matter, of the highest value, into a very small space.

The mortality is thus stated by Esquirol: "The highest mortality, for the two sexes, is between the ages of thirty and forty; that of the women is greatest between forty and fifty; that of the men between thirty and forty; from and after sixty years of age it is higher among women than men. It results that the mortality of lunatics is more precocious among men, and in advanced age infinitely greater among women." (Vol. i. p. 102.) From a comparison of different hospitals, he concludes the mortality in the different forms to be

| | |
|---|---|
| In Mania, | 1 in 25. |
| In Monomania, | 1 in 16. |
| In Melancholia, | 1 in 12. |
| In Dementia, | 1 in 3. |

Some idiots live a long time; but they seldom attain to more than thirty or forty years. The mortality of maniacs is greatest in the first two years of the malady; among the female patients (at the Salpêtrière) it is greatest in the first year.

It would scarcely appear credible that among the other extraordinary evidence given by medical men respecting insanity, they have on some occasions solemnly averred that it has no tendency to shorten life. The smallest consideration, one would think, would show that so serious a disorder of the nervous system must in general signally impair every function of life. Mr. Farr's statements and tables on this subject are, as usual, valuable, although perhaps somewhat intricate to those unaccustomed to statistical details. The following particulars are very intelligible.

"TABLE V. MORTALITY OF LUNATICS.

| Asylums. | Total Patients treated. | Living one year. | Deaths. | Ann. Deaths out of 100 living. | Deaths out of 100 treated. |
|---|---|---|---|---|---|
| Bethlem, St. Luke's, Stafford, York, Lincoln, Gloucester, and Hanwell —Lunatics................ | 19782 | 22295 | 2057 | 9.02 | 10.40 |
| General population, age 40-45, Sweden 1811-30. | ... | ... | ... | 1.50 | ... |

"The annual mortality among lunatics was 9 per cent.; the annual mortality of the Swedish population at the age of 40-45 was 1.50 per cent. (See *Annals of Medicine*, p. 359.) It need scarcely be added, that at this age the mortality of the Swedes differs inconsiderably from that of other European nations; *madness, therefore, increases the mortality sixfold.* But it is necessary to show that the mean age of lunatics does not *exceed* 40-45 years; as there are not observations sufficient to determine their mortality at different ages. The mean ages of 977 patients admitted in five years at Bethlem Hospital were in 1830, thirty-seven years; in 1831, thirty-five; in 1832, thirty-seven; in 1833, thirty-six; in 1834, thirty-six; so that 40-45 may be safely taken as representing the ages of the entire class—those above, as well as those below that central point." (p. 12.)

Other tables introduced by Mr. Farr, particularly one enriched by the accurate observations of Dr. Charlesworth, of Lincoln, marking the deaths as well as the admissions at decennial periods of life, tend to prove that the mortality increases, as the chances of recovery diminish, with age.

Too little attention has been paid to the *causes* of insanity; not by medical observers, but by the public; and yet the prevention of so terrible an affliction is even of more consequence than the possibility of its cure. An individual who has once been insane seldom wholly regains his social position; he is regarded with at least occasional suspicion; and a union with him is shunned by prudent families. Still, the early indications of a temperament prone to insanity are too little heeded, and proper preventive cautions too much disregarded. A valuable portion of Dr. Prichard's book is devoted to this subject. Many questions, he observes, connected with the theory of mental disorders, are yet and will perhaps always remain involved in obscurity, but an enquiry into the antecedents of insanity, and into the appearances disclosed by dissection, is accessible to us. We mention this as one enquiry, because the necroscopical phenomena are chiefly valuable as throwing light upon the physical origin of the disordered state of the mind, and form a part of that more general enquiry which comprehends the moral causes. The predisposing causes are still, we apprehend, the most important objects of study. Among these, Dr. Prichard considers the influence of constitutional predisposition, hereditary or original; sex; age; temperament; previous attacks of insanity and other diseases of the brain; and, lastly, education: being guided, throughout the enquiry comprehending these interesting particulars, by facts collected from various countries, and carefully considered.

That certain individuals, and certain families, are more prone to insanity than others, is a fact universally admitted on the most extensive testimony; and that when once it has arisen it is frequently transmitted, seems equally well established. M. Esquirol is of opinion that the children of parents in whom insanity declared itself at some period before their birth are more likely to become insane than those born before the appearance of the malady; an opinion of which we somewhat question the soundness. He is also of opinion that the disease is more frequently transmitted by the female parent than by the male. (Vol. i. p. 65.) When insanity has appeared in the family of both parents, it is reasonable to presume that the predisposition to it in the children is stronger. Among the poor, the hereditary cases constitute one sixth; but the proportion is still greater among the rich. (*Esquirol*, loc. cit.) He has now under his care the children of several patients whom he attended in the first years of his practice.

Out of 1380 patients admitted into the Middlesex Lunatic Asylum, it was ascertained that the parents or relatives of 214 had been insane; and in 125 of the cases no other cause could be assigned for the disease, Sir William Ellis says, than that it was hereditary. (*Treatise*, p. 42.)

Certain physical laws, obeyed by the constitutions of persons of the same family, seem to induce the malady at particular periods of life; in some families very early, and in others about the age of fifty. The effect of marriages between persons near of kin in producing a tendency to insanity has long been suspected. Accidental fright experienced by the mother during pregnancy is mentioned as a cause by Esquirol; and we have little doubt that the character and happiness of an unborn individual is often influenced by the general state of the mother's mind during her pregnancy. When insanity appears in several members of one family, it is commonly in the same form: the propensity to suicide has often been thus observed; and it is well known that a peculiar nervous temperament exists in some families, of which some of the members are mad, and some disposed to paralysis, chorea, epilepsy, or hysteria; some are melancholic, whilst some remain through life merely odd and *nervous*.

Whether women are less subject to insanity than men is a question of which different sides have been taken, on uncertain grounds, since the time of Cœlius Aurelianus, who maintained the affirmative. Dr. Prichard has compressed into two pages such satisfactory evidence of this not being the case, that we cannot do better than lay those pages before our readers. The question is by no means an unimportant one, in relation to the causes. The evidence is principally taken from M. Esquirol's report on the Royal House of Charenton.

"In the hospital of Charenton, during the years to which M. Esquirol's report has reference, the proportion of male patients admitted to that of female was three to two. This proportion is the result of peculiar circumstances, and is very different from that which obtains elsewhere, as well in France as in other countries. From a variety of public documents it appears that, in the great hospitals of the Salpêtrière and Bicêtre, an inverse proportion to that above stated holds in the numbers of the two sexes; male patients at the Bicêtre being to females in the Salpêtrière only as two to three. This, however, does not hold throughout France. It appears, from a great number of published and private documents which M. Esquirol has collected, that in the cities in the south of France the number of male is somewhat more considerable than that of female lunatics; while, in the northern departments, the number of females predominates in a greater degree; but that in all France the number of insane women is to that of insane men very nearly in the proportion of 14 to 11. From Spain the returns which M. Esquirol has been able to obtain are very defective: the result, which can only be looked upon as a presumptive one, since it rests on few data, and may hereafter be modified by more copious information, gives an excess of one fifth in the number of female lunatics compared with that of males. In Italy it appears, from the documents obtained, that the proportion is different; there being more insane men than women, particularly in the kingdom of Naples. In all Italy, 5718 male lunatics were reckoned, and 5067 females. In Holland and Belgium, according to the information given to the world by M. Guislain, it appears that the number of females in the lunatic asylums is much more considerable than that of males, being as 34 to 29; and this excess on the side of females is rather greater in the southern, or Belgian provinces, than in those of the north, or in the present Dutch kingdom. In Great Britain and Ireland, the proportion of male to female lunatics is as 13 to 12. In England, the number of men insane, compared to that of women, is more considerable than in Scotland and Ireland. This excess in the proportional number of male above that of female lunatics in England is greater, according to Dr. Burrows, in the higher than in the lower classes of society. M. Esquirol, who has cited this observation, adds a parallel remark in respect to the comparison of different orders of society in France; insanity being apparently more prevalent among men, compared with women, in the higher than in the lower classes.

"In the same memoir, M. Esquirol computed that, in the north of Europe, the proportion of male to female lunatics is greater than as above stated: he says that it is as three to two. In this are included the results obtained from reports of lunatic asylums in various parts of Germany, in Denmark, Norway, and Russia. Subsequently to the publication of this report by M. Esquirol, Dr. Max. Jacobi has given us extensive information on the state of lunatic asylums in the Prussian provinces on the Rhine. In the different establishments of that country, the total number of male lunatics, in the year 1824, was 1180, and that of females 835. The result nearly coincides with that deduced by M. Esquirol from other documents for the general proportion of Germany.

"In a later memoir, which appeared in 1830, M. Esquirol has given some new results from the statistical reports on lunatics in Norway, published by Dr. Holst. He reduces the proportion of male to female lunatics in that country to an excess of one sixth instead of one third part.

"In the United States of America, it appears from information given by Dr. Rush, by Portman of Massachusetts, and a report of the asylum in Connecticut printed in 1827, that the number of insane men is greater than that of female lunatics. Later researches have rendered this observation more precise. In the State of New York, Pennsylvania, and Connecticut, the proportion of males to females is very nearly as two to one.

"On summing up the results of his enquiries, M. Esquirol has shown that, in a sum total of 76,526 lunatics, confined, though not all at the same period, in asylums or hospitals in various parts of the civilized world, there were 37,825 males and 38,701 females. Thus the proportion of males to that of females is, a fraction being neglected, 37 to 38. This difference is so much the less considerable, as, in the general population, the number of males somewhat exceeds that of females; yet, small as it is, it is sufficient, as M. Esquirol observes, to refute the assertion of Cœlius Aurelianus, who supposed that women are less subject to insanity than men." (p. 162.)

We find Esquirol, however, stating (vol. ii. p. 138,) that *mania* is more frequent among men than among women. In men, he adds, the character of the mania is more violent and impetuous: consciousness of strength and the habit of command render them more audacious and more furious, more dangerous, and more difficult to restrain. Women are more noisy; cry and talk more; are more dissembling, and less easily confide in those about them.

Before the age of fifteen, insanity is a rare affection: it then becomes not unfrequent, and when middle age is attained, with its occupations and cares, affections of the mind are more common; being again less frequent (judging from the actual number of admissions) as age approaches. The attacks in early life are generally characterized by excitement, those of middle age more frequently assume the character of melancholy, and those of more advanced years of dementia; but these observations are liable, we presume, to numerous exceptions. Indeed, the proportion of individuals who, surviving till old age, then become insane, especially in the form of dementia, is considerable.

Among the predisposing causes of insanity, Dr. Prichard does not omit to make a few remarks on education; pointing out, especially, the evils of too great indulgence and a want of moral discipline, and an overstrained and premature exercise of the intellectual powers. Examples of the bad effects of the last-mentioned error are not rare; and it seems reasonable to suppose that a system of education calculated to inculcate habitual sedateness, and the restraint of violent emotions and passions, must tend to lessen the chances of attacks of violent mania. We are not, however, aware of any facts to which we could confidently point in proof of this not unreasonable supposition, although they might possibly be found in the statistics of insanity in the Society of Friends.

This subject is treated of by Dr. Prichard, in a section devoted to a consideration of the moral causes of insanity, and especially to the effects of religious impressions. It would appear that the number of insane in the Society amounts to nearly three in 1000; a very high proportion: but Mr. Tuke, who furnishes the details from which this proportion is computed, is disposed to doubt the data upon which the proportion of lunatics in England rests. Religious madness is very rare in the Retreat, or madness from intemperance, from disappointed affections, or domestic afflictions. Mr. Tuke also justly observes, that moral improprieties connected with mental peculiarities are more frequently stamped as insanity among quakers than in the world at large. Upon the whole, therefore, the presumption is, that there are fewer cases of insanity among them than occur in other sects.

The circumstance alluded to in a subsequent section on the Moral and Physical Causes of Insanity, that among savage nations, accustomed to indulge the most vehement emotions, mental disorders are considered to be rare; and the fact already spoken of, that women are not less prone to insanity than men, are not of a nature to encourage us to lay so much stress upon this class of causes as might at first sight appear reasonable: and, indeed, the impression upon our minds, made by some observation of the forms of insanity in individuals previously known to us, or whose previous habits and character we have been able to ascertain, is not that the majority of them were remarkable for great susceptibility to impressions, refinement of feeling, warmth of passions, or activity of mind, nor for having sustained great reverses or heavy afflictions. It is, however, to be stated, that the greatest number of inmates of the lunatic asylums of France consist of traders, merchants, and military men; persons who may be supposed to have been subjected to the extremes of hope and of disappointment. The various tables introduced into this section (Moral and Physical Causes) well merit the reader's attention; and they show, at least, that moral causes are more frequent than those of a physical kind.

Perhaps it will generally be found that private events, however appalling, and private afflictions, however severe, are less frequently productive of mental disease than those occurrences to which great publicity is given. The murder of the family of Marr, twenty years ago, in London, was productive of insanity in individuals, whom we have ourselves seen, in remote parts of the kingdom. The successes and the reverses, equally striking and dramatic, of the late emperor of the French, took possession of, and morbidly disturbed, many minds in many countries of Europe. Yet, even in these cases, it might be but the predominance of remarkable events in minds previously diseased, and prepared for usurpation by one idea. The history of France for forty years past has abounded in events so astonishing, or we might call them so romantic, that we can feel no surprise on finding that almost every change of the political scene has left its traces in the madhouses of Paris, as M. Esquirol says they have, from the taking of the Bastile to the last appearance of Bonaparte, and from that period to the present. Kings deposed, queens in obscurity, unrecognized dauphins, disregarded patriots, neglected commanders, emperors deposed, and chevaliers restored, abound in those institutions; just as, in our own establishments, are actually to be found zealous reformers in church and state, and clergymen alarmed to madness by the dread of Roman catholic ascendancy. These circumstances fix themselves morbidly on weak and wandering intellects, and utterly disorder them. Real and pretended discoveries in science have often the same effects: electrical experiments, phantasmagoria, animal magnetism.

The causes of melancholia are forcibly depicted by Esquirol, (vol. i. p. 422.) He enumerates among them the air of marshes, and, during the prevalence of particular winds, hot and dry countries. Melancholia is said to be frequently produced in dry and burning atmospheres, which exalt the sensibility and render the passions vehement. Such was found to be the case in Egypt and in Greece, according to Aretæus, Bontius, Prosper Alpinus, and Avicenna; and modern travellers have made similar observations in Asia Minor, Upper Egypt, Bengal, and the coasts of Africa. In the autumn of 1818, the summer of that year having been unusually hot in France, cases of melancholia were more numerous than usual; but the influence of the seasons seems by no means firmly established. Cases of melancholy appear, however, to be most frequently cured in the spring.

Children have been observed to become melancholy and delirious in consequence of jealousy, excited by the attentions of those to whom they were attached being given to others: they have become pale, and have sunk into fatal marasmus. Melancholia, sometimes of a religious kind, sometimes arising out of disordered passions, is observed at the period of puberty. In adult age, many causes excite it, both in men and women. In old age it is rare. The melancholic temperament is well known to be often associated with great talents, and great powers of application, for good or evil. Professions which excite the imagination and the passions are found strongly to predispose to it. Musicians, poets, but, above all, actors, are very liable to it. Errors in diet, and especially excess in drinking, directly tend to produce it; and Voltaire expressed an important truth when he observed that constipation exercised a disastrous influence on the determinations of the great. Impairment of the functions of the skin has been thought to act as a cause; and sometimes the retrocession of eruptions. But disorders of the passions and affections are, according to M. Esquirol, the most frequent of all the causes of melancholia. The causes of this, as of other forms of mental disorder, do not always exercise their primary influence on the brain: their first effects are often produced on the abdominal viscera, and pass on to hypochondriasis, and from that to melancholia. Instances are mentioned in M. Esquirol's work, and have been the subject of separate observations made by him, in which the particular fancy and supposition of the patient in these cases arose from actual local disease; a point of some practical importance, although we apprehend its existence has scarcely yet been recognized in the greater number of lunatic asylums. Ulcers in the throat; chronic peritonitis; cancer of the stomach; gangrene of the transverse arch of the colon, have occasionally existed as causes of the phenomena from which hypochondriacs drew false conclusions as to the presence of living creatures in the abdomen or the throat.

The following tables, quoted by Dr. Prichard, illustrating the proportion of cases of insanity in the married and unmarried, are curious, and may supply matter for useful reflection.

"Total number of male lunatics, imbeciles, and epileptics in Bicêtre, January 1, 1822 . . . . . . . . . . . . 764

"Total number of females in the Salpêtrière, at the same date . . 1726

"They are distributed as follows:

| | Females. | Males. |
|---|---|---|
| "Unmarried . . . . | 980 | 492 |
| Married . . . . . | 397 | 201 |
| Widowers and widows . . | 291 | 59 |
| Divorced . . . . . | 5 | 3 |
| Not noted . . . . . . | 53 | 9 |
| Total . . . | 1726 | 764 |

"In Germany the proportion of unmarried to married persons is much greater, as may be judged from the following numeration, which I take from Dr. Jacobi's *Statistik.* The total numbers of 1180 male lunatics and 835 females are thus distributed:

| | Females. | Males. |
|---|---|---|
| "Unmarried . . . . . | 599 | 974 |
| Married . . . . . | 156 | 176 |
| Widowed . . . . . . | 80 | 30." (p. 185.) |

Dr. Prichard observes, that if the disproportion displayed in these tables were found generally to exist, it would constitute a leading feature in the history of mental derangement; but that the reports of most hospitals are not kept with sufficient accuracy to determine the fact. The reader can scarcely fail to perceive that Dr. Prichard's references are almost invariably made to foreign authorities and foreign documents; and to interpret this circumstance into a tacit condemnation of our own establishments, if not of our own writers, too much, we fear, deserved. It is, indeed, surprising how little our large lunatic establishments have contributed to the general knowledge of insanity. The chief care seems to be devoted to the mere safe-keeping of the insane; and statistical information, and even the cure of the disorder, appear to be comparatively overlooked. Lunatic asylums, instead of being hospitals, become show-places.

Among the moral causes of insanity, Dr. Prichard has discussed with great fairness and ability the comparative prevalence of insanity in different religious sects; and especially in Catholics and Protestants. The result seems to be, that strong enthusiasm, wild preaching, and vivid and fearful impressions produce madness in the followers of all sects.

The disregard of religion, prevalent selfishness, and want of domestic affections, are noticed by M. Esquirol as so many causes of disordered mind in his own country; and to these he adds a system of education in which the mental faculties are more cultivated than the affections of the heart. (Vol. i. p. 50.) These causes appear to be but too general in all countries. An important lesson may be gathered from the fact stated by Esquirol, that, of the moral causes of insanity, the most frequent are pride, fear, alarm, ambition, reverses of fortune, and domestic disquietudes (*chagrins domestiques*). Moral causes, he also remarks, are more frequent than physical causes of insanity.

After mentioning, among the physical causes of insanity, injuries of the head, Dr. Prichard alludes to some of those extraordinary examples in which the faculties were improved by such accidents. One of the innumerable points on which the wit of the opponents of phrenology has occasionally been exercised is the possibility of this occurrence; Dr. Gall, we believe, having related the case of an idiot who became very reasonable after falling out of a two-pair-of-stairs' window. It was even recommended that a hint should be taken from such an example for the enlargement of our curative resources, and that projection from the windows of upper stories should be added to other therapeutic agents for supplying the deficiencies in like portions of the human frame. These witticisms are shown by cases cited by Dr. Prichard, who abjures phrenology, to be quite superfluous: he says he has been informed on good authority that there was some time since a family, not far from Bristol, in which there were three boys, all considered as idiots; but that one of them having received a severe injury of the head, his faculties began to brighten, and he became a man of good talents, and practised as a barrister; his brothers, less fortunate, being still idiotic or imbecile. Like cases are also quoted from Van Swieten and Haller.

Exposure to the ardent heat of the sun has been a frequent cause of madness; and exposure to the heat of a fire has produced similar effects on cooks. The suppression of eruptions and discharges has long been noticed as an occasional cause; and Dr. Prichard adds, that he has seen some cases of mania supervening on inflammatory rheumatism which had subsided; "cases of very acute inflammation affecting the large joints, which had been reduced by too profuse bleedings, a measure which in this disease also gives rise to metastasis."

We find several details of interest connected with the subject of climate and season in M. Esquirol's treatise. The temperate climates, in which changes of the atmosphere are frequent, are the most productive of insanity. Excess of cold in Russia, and of heat in Egypt, produced many cases in the French army. The atmospherical commotions about the equinoxes are found to excite lunatics; they become more noisy, and demand more care. The admissions to the Salpêtrière are most numerous in May, June, and July, and least numerous in February and March. Some lunatics are so only in summer, and some in winter. When an attack shows itself in spring or summer, if it does not end promptly, it is commonly prolonged to the winter. The monomania and mania of autumn only end in the spring. Summer most favours the cure of dementia. Cures which take place in the hot season are rare, but of a permanent character. M. Esquirol has not been able to satisfy himself with relation to the question of the moon's influence. As regards the *epidemic* character of madness, he observes that there certainly are years in which, independently of moral causes, madness seems all at once to appear in a great number of individuals. (Vol. i. p. 25.)

The abuse of stimulating liquors is a frequent cause of insanity among the English, the Germans, and the Americans; but rare among the French. Opium has sometimes had the same effect. There is, as regards both opium and mercury, a peculiar idiosyncrasy in some individuals, by which they are roused to extreme irritability, bordering on mania, by these medicines; which, if the medicine is persevered in, may, and which we doubt not in some cases has, gone on to mania. Insanity in consequence of sensual vices is probably more common in France than in the countries above mentioned as so unfortunately distinguished by excess in spirituous liquors. We find frequent allusion in Esquirol, and indeed in most of the works before us, to the serious consequences of manustupration. It is possible, we think, that too much stress is laid upon this habit as a *cause*, the actual circumstances of lunatics in confinement being considered. We must remark, that Sir William Ellis's attempt to guard his general readers from details of this kind by putting them altogether into an appendix, is likely to be as unfortunate in its result as the very similar one of the editors of certain Classics for the Use of Schools, who omitted objectionable passages in the text, but carefully collected them all at the end of each volume. Intestinal irritation has already been alluded to; and the various states of the uterine system which lead to insanity have attracted general attention. An attempt has been made to specify the state of the intestinal canal which is often connected with mental disease; and Dr. Prichard's opinion concerning it is contained in the following passage.

"The state of the intestinal canal to which I allude is itself much more frequently of an inflammatory nature than it has generally been imagined, or at least than it was formerly supposed to be. In that condition of the canal which gives rise to costiveness alternating with diarrhœa, and accompanied with indigestion, flatulence, and eructations, anorexia and nausea, transient but often acute pains in the hypochondria, livid and yellow suffusions of the skin, viscid secretions in the mouth, or redness of the fauces and palate, with a glazed and dry surface, the whole train of symptoms often depends upon a low degree of chronic inflammation in the mucous membrane of the intestinal canal; and this is perhaps a frequent, if not an ordinary state, in those cases in which disorders of the nervous system supervene on complaints of the stomach and bowels. This form of disease has been described by Dr. Ferriar and several other practical writers; but it is to M. Broussais that we are indebted for a more ample development of its pathology.

"The enteric disorder, which lays the foundation for maniacal symptoms, as well as for other affections of the nervous system, is the result in different instances of various and very diverse noxious causes. The most frequent is excess in the use of stimulant and indigestible food. Too great indulgence of the appetite among the more opulent, and among the lower classes long-continued constipation, unwholesome diet, the use of salt provisions, exposure to cold and want, or neglect of warm clothing, give rise to diseases of the same description." (p. 206.)

Such is probably the explanation of many of the cases of insanity which occur in quiet parts of the country, as in agricultural districts, where insanity is by no means an uncommon affection: but we have little doubt that there are many shades of intestinal irritation, most seriously affecting the brain, short of the inflammatory condition. In towns and cities, especially in those in which the customary diet is stimulating, as it certainly is in Paris, the irritation may more frequently assume the inflammatory or sub-inflammatory character.

It would, however, be an error to ascribe all cases of insanity to intestinal disorder, in which marks of intestinal disorder existed during life or are found after death. Inflammation and ulceration of the mucous membrane of the intestines no more prove, as M. Esquirol observes, that that membrane was the seat of the origin of the disorder, than they prove, in phthisis, that the disorder begins there. Lunatics are obnoxious to many kinds of chronic inflammation; they become scorbutic; they become phthisical: we must expect, therefore, to find the intestinal mucous membrane diseased. (Vol. i. p. 87.) In three eighths of the cases, however, the same authority admits that the patients *die* of diseases of the abdomen; in two eighths, of diseases of the chest; and in two eighths, of encephalic affections. (p. 104.) These calculations are the result of the examination of about 600 cases after death. We must express our belief, founded in part on the Reports of lunatic establishments, that the corporeal ailments under which the unhappy patients languish and die are very often overlooked, and consequently are not met by proper treatment. The establishments, we have observed, are made a kind of show-houses: they should be hospitals for the complicated diseases which involve the functions of the mind. We grant that the mental malady may often be but the first sign of that total impairment of the frame which phthisis, or hydrothorax, or scorbutus, or paralysis, or marasmus afterwards more plainly declare; but we suspect there are cases in which, if the life of the patient were preserved through some of the maladies supervening on the mental disorder, the mind would be found to be restored, and the malady to be critical. With the present management of lunatic hospitals, these conjectures can neither be verified nor refuted. In many of them medical aid is considered to be nearly superfluous; and in some we are informed that the appointment of physicians in ordinary has been, if not rejected by the governors, at least subjected to grave debate, as if the county asylum were no more than a supplementary county-jail.

Pinel observed that lunatics are liable to sudden and fatal attacks of apoplexy, particularly in winter. It is most common in the old. All at once the most violent fury or delirium ceases, and in a few moments the patient dies; as if all the forces of life were exhausted. (*Esquirol*, vol. i. p. 107.) In one case, that of an emaciated man of seventy-two, there had been continual delirium with agitation for three months: the patient, on awaking, very calmly asked his servant for his snuff-box, took a pinch of snuff, and expired. Putrefaction of the body proceeded rapidly, and no alteration was found within the cranium.

M. Esquirol maintains that insanity may be either continued, remittent, or intermittent. In the continued form there is an acute stage, with concomitant symptoms; then a chronic stage of simple delirium; and a third stage, in which the attack declines. Remittent cases present remarkable anomalies, both as to the character and duration of the complaint: a patient may be three months melancholic, and three months maniacal, and then four months or more in a state of dementia; and so on successively, with more or less regularity. Some maniacs are only violent at certain times of the day, or on certain days or seasons. Intermittent cases may be quotidian, tertian, quartan, monthly, annual. (Vol. i. p. 79.) He thinks, also, that the doctrine of crises is applicable to cases of insanity; and that a cure unmarked by a crisis is not to be depended upon. Sometimes, as he expresses it, the disease terminates by resolution, and sometimes in a predominance of the absorbent system; the patients become fat, and the delirium disappears. If the delirium continues, the obesity is a sign of dementia. In other cases, emaciation advances almost to a fatal point, and then recovery takes place. These terminations M. Esquirol considers to be critical. Many cases are resolved by fever. The appearance and the disappearance of the catamenia often prove equally critical. Among the cutaneous diseases which by their appearance put an end to madness, M. Esquirol mentions the itch, according to the opinion of Hippocrates. Persons of acute sensibility well know, we presume, what it is to go through the actions of crying, and yet not to shed a tear. Maniacs are subject to this; and, when they do shed tears, a cure sometimes follows. A transpirable state of the skin, restored by warm baths or by the return of spring, is often very serviceable. Several interesting examples are mentioned of cures effected by moral impressions: in one of the cases, a man, on his way to drown himself, was attacked by robbers; he defended himself stoutly, and got rid of his madness.

In the fifth chapter of Dr. Prichard's treatise the very various morbid appearances detected in the head in cases of insanity, by Morgagni, Greding, Haslam, Pinel, Esquirol, Georget, Bayle, Calmeil, and Foville, are clearly reviewed; and although they do not, perhaps, conduct the reader to very positive conclusions, certainly deserve his attentive consideration. The results of M. Foville's examinations are not yet very familiar to English students. In acute cases, he has observed intense redness of the surface and of the substance of the grey matter, without any adhesions of the membranes to it; such adhesions being, he thinks, a characteristic of the chronic cases, connected with their incurability. In the chronic cases, the cortical substance, to a certain depth, was found firm and dense, constituting a distinct lamina; which, when torn off, left the remainder of the cortical substance red, soft, and mammillated, somewhat resembling granulations: in these cases the surface of the cortical substance is very pale. The volume of the convolutions is either natural or diminished. There is sometimes a real atrophy of the convolutions, which is frequent in the frontal regions of the hemispheres; and a chasm filled with serosity occupies the place of the absorbed substance. The diploe disappears in these cases, the external lamina of the cranium approaches the internal, causing a superficial depression on the head. Softening of the cortical substance is also a phenomenon observed in chronic cases; an equal softening of the whole cortical substance; and this is sometimes coexistent with increased hardness of the medullary portion. It would seem that these appearances belong to cases of the last degree of dementia, with general paralysis and marasmus. The colour, density, and texture of the white substance also are not unfrequently altered in the brains of lunatics: it is sometimes more or less injected: sometimes of a splendid white colour, and increased in hardness; an effect which M. Foville tries to account for by supposing that each cerebral fibre has contracted adhesions with the surrounding fibres. Sometimes the brain is full of serous fluids; and sometimes limpid fluid is collected in numerous small cavities, from the size of a millet-seed to that of a nut, and supposed to be the sequelæ of extravasations.

The changes which have been observed by M. Foville in the membranes are, in acute cases, injection of the pia mater, the arachnoid preserving its natural aspect: in chronic cases, the arachnoid is opaque and thickened; granulations and pseudo-membranes are formed on its surface, and serosity is effused into the cellular tissue of the pia mater and the ventricles.

These appearances, M. Foville remarks, are all indicative of inflammatory disease, affecting the brain itself; the changes in the membranes being only accidentally complicated with it; an opinion quite opposite to that maintained by M. Bayle, who considers insanity to be the effect of inflammation of the membranes. The few consecutive necroscopical observations made in our own lunatic establishments, if they do not entirely countenance the opinion of the invariable existence of the appearances described by M. Foville, are at least insufficient to contradict it; although it is possible that the desire to maintain particular views may have exercised an unconscious influence on M. Foville's powers of observation. The whole history of medicine tells us to distrust the most those systems which appear the most imposing by their uniformity and simplicity. When will our large public institutions for the insane assist in the elucidation of these questions?

The most constant changes observed in connexion with mental affections are found, according to M. Foville, in the cortical substance of the brain; and he considers the view of M. Calmeil, who ascribes the paralysis to these changes, as incorrect; the paralysis being always attended by some alteration of the white substance, superadded to the cortical change.

"A very remarkable case, which occurred in the clinical course of M. Esquirol, in 1823, affords strong evidence in favour of M. Foville's argument. The cerebrum of an idiot displayed the grey substance of both hemispheres in the last stage of atrophy and disorganization, while the white portion of the brain remained perfect on one side. In this person the intellect had been entirely defective, but the muscular power on one side only had failed. From this and similar observations, M. Foville concludes that the function of the cineritious portion of the brain is essentially connected with the intellectual operations, and that of the fibrous or white structure with muscular action." (p. 225.)

The case here adduced in support of the opinion is extremely interesting; but the opinion itself is by no means a new one. M. Foville's principal inferences, however, are, that morbid changes in the cortical substance are directly connected with intellectual derangement, and changes in the white substance with disorders in the motive powers.

In cases of puerperal madness, the paleness of the brain, and the absence of the changes above mentioned, have led M. Foville to consider this form of mental disorder to be the result of some deeply-seated disease of the uterus or abdomen. There can be little doubt that the contradictory evidence given by different observers of the appearance of the brain in insanity is, in part, to be accounted for by the existence of cases in which the affection of the brain is merely functional and sympathetic; the primary disease being in some other organ, especially in some of the abdominal viscera.

We consider the observations of M. Esquirol, in relation to the morbid appearances, and to the conclusions to be drawn from them, as highly worthy of being remembered. The various states of the cranium and the brain compatible with integrity of the cerebral functions should be scrupulously ascertained; and the appearances resulting from or belonging to concomitant maladies be carefully distinguished from those which belong to the mental affection. The existence of organic lesions of the brain is declared by symptoms distinct from the madness: chronic inflammation produces compression and paralysis, and paralysis results from cerebral hemorrhage: tubercles, tumours, and softening of the brain have their peculiar symptoms, which cannot be confounded with mental alienation. Moreover, the sudden and instantaneous relief experienced in some cases of madness is not to be forgotten. Two circumstances, also, mentioned by Esquirol, when enumerating the morbid appearances actually found in different cases, ought ever to be borne in mind; namely, that, in many dissections of lunatics, no alteration of any kind has been found, although insanity had existed for many years; and every part of the brain has been found altered, suppurated, destroyed, without chronic lesion of the understanding. (Vol. i. p. 113.)

The maniacal form of insanity is, M. Esquirol says, rarely fatal. The patients do not die of the cerebral affection, but of typhus fever, phthisis pulmonalis, and epileptiform convulsions. They die suddenly, as if the sensibility necessary to life was exhausted. In a case previously mentioned, in which death took place in this manner, no cerebral lesion was found after death; and the same was observed in the case of a young woman who was accidentally killed in a state of recent and furious mania. Sometimes the brain and its membranes are found uninjured when the disease has lasted some years. When a case is watched during life, he thinks the epoch in which the cerebral lesion commences may be known by the symptoms. When mania has existed long, he is of opinion that the weakness of the last days of life disposes to local inflammations. Upon the whole, he concludes that, notwithstanding the labours of MM. Foville, Calmeil, Bayle, and Guislain, pathological anatomy has not yet declared the organic reason of mania. "Thirty years ago," says this veteran observer, "I should have written willingly on the pathological cause of insanity: I will not now attempt so difficult a labour, such are the uncertainty and contradiction in the results of the examinations of the bodies of lunatics after death up to this day. But I add, that modern researches permit us to hope for more positive, clear, and satisfactory notions." (Vol. ii. p. 181.)

Severe diseases of the lungs, obscure or unsuspected during life, appear to be very common in cases of insanity: even cavernous excavations, when no expectoration has been observed. Diseases of the heart are frequent attendants on insanity; but the nature of the relation of these, as well as of the pulmonary affections, to the insanity, is not clearly established. They are probably not to be looked upon as causes. Of abdominal affections existing in cases of insanity, inflammation of the mucous membrane of the alimentary canal seems to be the most common. M. Esquirol, some time ago, described a displacement of the colon, which he had observed in several examples of melancholia. The colon, in these instances, had assumed a perpendicular position, its left extremity descending to the pelvis behind the os pubis. If the state of the liver has often a close connexion with insanity, as has been so long and generally considered, this organ is, at least, seldom found to have undergone structural change in such cases. A great number of melancholic patients are found to die of phthisis pulmonalis; many die of abdominal diseases; but organic alterations in the intestines are rare in them. (*Esquirol*, vol. i. p. 464.)

As insanity appears in some instances to arise from direct disorder in the brain, without structural change, and sometimes from evident disease of that organ; as in other cases it appears to be occasioned by the disease first affecting other organs, as the liver, intestines, uterus, &c., and secondarily affecting the brain; and as, in addition to these cases, there are probably others in which, the first impression being made on the brain, a secondary effect is produced on the liver, intestines, uterus, or other organs, from which a reflex action and more intense disorder is occasioned in the brain; it cannot occasion surprise to find what ingenious disputes have hence arisen among pathologists as to the first cause of insanity: some maintaining it to be a disease of the mind, some that it is always idiopathic, and others that it is an entirely sympathetic disorder; the source of which has furnished yet further subject of controversy.

Dr. Prichard maintains that, in a great proportion of cases, the whole train of phenomena depends upon inflammation of the brain and its membranes; and that in the rest there is every reason to believe that a condition of the brain is primarily or secondarily induced which has a tendency to put on an inflammatory character; and he observes, that, in cases of long duration, the disorganization of the brain which renders recovery impossible, or generally precludes it, is produced by inflammation. These opinions have, of course, an important bearing upon the treatment.

TREATMENT OF INSANITY. We observe in M. Esquirol's volumes occasional regrets expressed that so little effort is made to make lunatic asylums places of instruction for pupils. Mr. Farr also alludes very strongly to this subject. Of the practicability of this, under certain obvious regulations, no doubt seems to be entertained by him; and a similar opinion is given by Sir W. Ellis, and by Mr. Browne, whose work on Lunatic Asylums we noticed in a former Number. This subject was earnestly advocated, but without effect, ten years ago, by the professor of medicine, on the establishment of the medical school of the London University. Up to this period very little has been done to facilitate the acquisition of a knowledge of the practical details of the very serious class of mental disorders; and, although the interests of the public imperiously demand that medical practitioners should be furnished with more exact notions and indications of treatment in the various cases received into asylums for the insane, there has not yet been found sufficient moral courage in the managers of any of the great public institutions, or sufficient enterprise in any medical school, to establish clinical instruction on a scale sufficiently extensive, or a plan sufficiently systematic, to diffuse a general acquaintance with the phenomena and the therapeutics of insanity. In this state of things, the practical experience of those who have had the care of many lunatics, and who communicate the results of their practice to the public, ought to be attentively considered.

We should first, well observes M. Esquirol, endeavour to ascertain if there are any urgent indications to be fulfilled. When there is lively excitement and plethora, bleedings, and tepid baths in which the patient is kept a long time, cooling drinks, laxatives, light diet, and sometimes counter-irritants applied to the skin, almost always produce a remission, and sometimes a very marked intermission, in eight, fifteen, twenty-one, or thirty days. Time is then afforded to consider and combat the causes of the disorder. If hemorrhages which were customary have been suppressed, if an ulcer has dried, or a cutaneous affection disappeared, previous to the outbreak of mania or monomania, the reappearance of these will almost certainly cure the mental affection. (Vol. i. p. 145.) If this effect should not follow, we may then have recourse to *empirical* treatment.

Dr. Prichard arranges his practical observations under the two natural heads of the Therapeutical and the Moral; the latter being a term commonly used by writers on disorders of the mind, to express a combination of mental with moral means of forwarding the cure. For the sake of order, we shall arrange our observations according to this unexceptionable division; which is also that adopted by Dr. Ferrarese.

*Therapeutical Treatment.* The indications belonging to this head, as laid down by Dr. Prichard, differ in nothing from the general indications of cure in all other diseases; the first object being to restore, if possible, the diseased organ to a state of health; an indication chiefly, but not exclusively, applicable to the acute stage and early period of insanity. The second indication "is to restore and maintain, as far as it can be done, a healthy condition of the physical or natural functions, and to obviate or remove disorders in other parts of the system, which may be connected or coincident with the diseased condition of the brain." (p. 275.) This indication is evidently in some degree involved in the first.

In discussing the different particulars comprehended in the therapeutic treatment, Dr. Prichard manifests his usual excellent judgment, reviewing each candidly and deliberately, and expressing his opinions with calmness and discrimination. He assumes, as we have before observed, that the condition of the brain usually connected with disordered manifestations of mind is one of excitement passing on to inflammation; but he cautions the reader against therefore concluding that the disease is certainly to be cured by antiphlogistic measures (p. 251); inflammatory excitement, he observes, being but a part of the disease, and not wholly constituting it. In many patients, an active adoption of antiphlogistic measures, whilst it would not subdue the disorder, would exhaust the powers of life. This is one of the points in the treatment of the insane on which we have repeatedly observed medical men at fault: nor can we wonder that they should be so: nothing but a larger acquaintance with cases of insanity than usually falls to the lot of private practitioners seeming sufficient to show, beyond doubt, that cases presenting, as it would seem, very manifest signs of arterial excitement, especially affecting the brain, will not be benefited by,—will not even bear without exasperation or unfavorable result of some kind,—the treatment which vascular excitement seems plainly to require. The clinical practice of a well-managed asylum could alone throw full light on this and on many other parts of the treatment; for there are cases of insanity, particularly of recent date and in vigorous persons, in which antiphlogistic measures are found to be of immediate and important service. The opinion of Pinel, that bleeding increases the tendency of the disorder to pass into dementia is, Dr. Prichard remarks, deserving of attention; but he quotes the testimony of Esquirol in favour of moderate bleedings in plethoric cases, and particularly by a few leeches applied at a time, with cold applications to the head. Dr. Haslam, Dr. Rush, and Joseph Frank, were much warmer advocates for bleeding; and M. Foville, observing that adhesions are very common in chronic cases, and very rare in acute ones, and, when they have once taken place, are incompatible with the return of reason, concludes that bleedings, general or local, are of great importance in the greater number of recent cases; in which opinion Dr. Prichard coincides. (p. 258.) His observations on the particular cases that call for bleeding are especially deserving of every practitioner's reflection. After specifying these cases, Dr. Prichard does not conceal the remarkable fact that, in the Gloucester Lunatic Asylum, under the superintendence of Dr. Shute and Mr. Hitch, "the use of the lancet, leeches, cupping-glasses, blisters, drastic purgatives, the practice of shaving the head, are totally proscribed;" and yet that recoveries take place in a large proportion, and *no cases* of sudden apoplexy or hemiplegia have occurred. (p. 261.)

Sir William Ellis makes some important observations on this head: he says that many of the patients received into the hospitals as recent cases have been previously bled to excess, so that the constitution cannot rally, and a much greater mortality takes place in the recent cases than among the old. There is a greater probability of cure, he thinks, in cases in which the cause is permanent, if the attack is allowed its own course, than when the powers of the constitution are wasted by excessive depletions. (*Treatise*, p. 150.) Except in actual phrenitis, and in cases arising from physical injury, he scarcely thinks free bleedings allowable. In cases acknowledging a moral cause, he is convinced that the patient generally requires to be supported, although local bleedings may be very serviceable. (p. 168.) He says also, that, in many cases of insanity manifested in particular propensities, he has found a greater degree of heat in the scalp covering the region of the brain assigned by phrenologists to such propensity; and the patients have so generally complained of pain in that locality, that, when they are silent, he is governed by the character of the existing derangement in the local application. This kind of evidence is open to obvious objections, and yet may be perfectly true. A more important observation made by Sir William Ellis is, that by small local bleedings, promptly had recourse to, on the appearance of the symptoms of a renewed attack, in chronic cases, a patient, who would otherwise suffer for months, may often be relieved in a few days. (p. 249.)

In the acute stage of mania it is best, according to Esquirol, (vol. i. p. 140,) that the patient should be in a cool and dark place, as recommended by the ancients; but, in the chronic stage, he agrees with Pinel that they should be allowed free scope for their activity in the open air. When the disorder has come on in a hot climate, a return to a cooler increases the chances of recovery, and *vice versâ*. The clothing, especially of the melancholic, should be of woollen, even next the skin; and frictions of the skin are found useful. Esquirol recommends a mattress, a bolster, and a pillow of horse-hair: the coverings should be light, and the head uncovered.

Shaving the head once or twice a week is spoken of with approbation by Dr. Prichard. The cold shower-bath is chiefly applicable to young persons: in cases of much excitability, the reaction is an objection to its employment; and there is danger, in old cases with a disposition to congestion, of producing paralysis. Pouring small quantities of cold water on the head until it covers the body and produces shivering, has sometimes been useful. Placing a patient in the warm bath, with ice constantly applied to the head, has also been of much service; but the patient requires to be put into the bath more than once a day, and for two or three hours each time. Warm or tepid bathing is often of much use to those patients in whom the skin is cold and the circulation languid. By all these methods of bathing, sleep is often obtained, and the agitation strikingly diminished. Dr. Ferrarese urges the necessity of keeping the patient a sufficient time in the warm or tepid bath; and M. Esquirol seems to approve of the use of the tepid bath in meager, nervous, and very irritable subjects, prolonged for several hours: cold should be applied to the head at the same time, either by means of cloths dipped in cold water, or by cold water contained in a bladder. Sir William Ellis recommends pounded ice in a cap of waterproof cotton. The cold bath agrees very well with young and robust subjects, who suffer much from heat. For subjects enfeebled by vicious practices or long solicitude (*chagrins*) the plunge bath and cold affusions are suitable. Several cases of mania are related by M. Esquirol, (vol. ii.) in which affusion was singularly serviceable. Pinel proscribed, and M. Esquirol has never employed, the "bath of surprise;" not only conceiving, probably, that little good was to be expected from throwing an unhappy lunatic into water unexpectedly, and allowing him to be nearly drowned, but for the more serious reason, it would seem, that he knows it to have been fatal. (Vol. i. p. 147.) He avows, in fact, that he does not think it preferable to throwing the patient out of the third story, on the principle which we have already alluded to.

We never fail to find M. Esquirol calm and judicious in his estimation of remedial means. Although he speaks of the *douche* as refreshing and sometimes useful to lunatics who are young, strong, and active, and particularly where there is pain of the head, calming their fury, and reducing them to obedience; he also points out that, when received on the head, it exercises a sympathetic action on the epigastrium, causing pain and an inclination to vomit; and that, after its action, the patients are pale, and sometimes *yellow*. It should never be exhibited after a meal; and the first passages should be cleared before it is taken. It should not be continued more than a few minutes; nor should its application, which sometimes produces serious accidents, ever be intrusted to servants.

In some cases of stupor, small quantities of water thrown upon the face have occasionally been serviceable.

The use of blisters and counter-irritation by the tartar-emetic ointment, and of setons and issues, is more limited than might be expected; being chiefly confined to the chronic forms of the disease, to monomania accompanied by stupor, and to cases characterized by torpor and insensibility. In cases of paralysis, with a tendency to coma and lethargy, Dr. Prichard has found these remedies decidedly advantageous. He considers setons best adapted to disorders of a chronic form; "but when there is great intensity of disease, and a state of the brain threatening a fatal increase, issues made by a long incision in the scalp over the sagittal suture are particularly useful." Dr. P. has found this means more beneficial than any other in stupor and dementia following attacks of apoplexy, or paralysis, or severe fevers; and he would adopt it in the general paralysis of insane persons, although, as might be expected in a disease of which we have seen that M. Esquirol considers the course rapidly progressive, not with very sanguine hope of benefit. We do not find M. Esquirol speaking hopefully of setons, or even of the actual cautery applied to the back of the neck in these cases, (vol. i. p. 154;) although this method has sometimes been used with success in furious mania. The cases in the second volume (p. 216,) would incline us to ascribe much of the good effect of this means, when it succeeds, to the fright which it occasions. In all cases of insanity, the moral effect even of physical remedies requires to be taken into the account.

The utility of purgatives has been long established. They are, however, sometimes of very little service; and, in particular states of the intestinal canal, hurtful. One of the critical terminations of insanity, occasionally occurring, is diarrhœa.

In the management of an insane person, a practitioner must now and then expect to find opposition to medicine, in consequence of the patient believing himself to be in much better health than usual. In these cases, Esquirol observes that if some medicine is given without the patient's knowledge, and which excites pain and evacuations, it causes him to become more anxious about himself, and makes him also more docile. He has not found external frictions with purgative substances effectual; and this would either seem to have been commonly the case, or this otherwise convenient method of employing such medicines in cases of lunacy has not attracted much attention.

Somewhat conflicting testimony exists respecting the good effects of emetics. It has been, it seems, for many years, the practice in the Bethlem Hospital to give the curable patients four or five emetics in the spring of the year; in Dr. Haslam's opinion, without benefit. Dr. Cox strongly recommends them; and Dr. Wake has found them very efficacious in the York Asylum. It is obvious that the use of such remedies should not be indiscriminate; but they may probably be found advantageous in cases of melancholia with stupor, and even sometimes during furious excitement, in which their exhibition is said to have been found to be followed by tranquillity and by sleep. In cases of violent excitement, six or ten grains of tartarized antimony are required to produce vomiting; but Dr. Prichard recommends that moderate doses be begun with, combined with ipecacuanha. Nauseating doses of antimony are certainly often serviceable in controlling maniacal excitement. In violent cases, Sir William Ellis speaks highly of the effects of half a grain of tartar emetic given every three hours, combined with sulphate of magnesia, until copious vomiting and evacuations are produced. This is rather severe practice, however. We are still less inclined to think, with Sir W. E., that the nauseating effect of antimony is advantageous in melancholia; the miseries of which it can scarcely lessen.

Where there is considerable excitement of the heart and arteries, digitalis, so much extolled by Dr. Cox, and on the continent by Müller and Guislain, will probably be productive of relief. In the Hanwell Asylum it has been found useful as a means at once of reducing the circulation and increasing the urinary secretion, to give fifteen drops of antimonial wine, and half the quantity of the tinctures of digitalis and of squill, with fifteen of the spiritus ætheris nitrici, and ten grains of nitre, three or four times a day.

We believe that opium and other narcotics have, during late years, been used in some of the asylums in this country, with a degree of boldness of which the profession in general is not at all aware; and, if we are rightly informed, with proportionate success. Dr. Prichard, whilst he admits that opium is in some cases decidedly useful, advises caution in its employment; and he considers it *generally* injurious when the vascular system of the brain is overloaded with blood. (p. 269.) He has seen apoplexy, in such a state, supervene on its administration in a moderate dose: but, he adds, "there is a condition of the living body, produced by long-continued excitement or stimulation, when the powers of reaction are beginning to be exhausted, and what the Brunonians term indirect debility ensues, in which the sudden abstraction of stimuli gives rise to extreme prostration;" and in this state, as in delirium tremens, he thinks that opium may be beneficial, "by sustaining the vital actions in a certain degree of energy until the restorative powers of the constitution can come into play, and exert their usual influence." (p. 269.) In cases of long-continued restlessness it would appear clearly indicated. Moderate doses are not, we think, usually very satisfactory in their results. Van Swieten, Cullen, Darwin, and Klieber of Berlin, are quoted as having given it in large quantities; but Dr. Prichard is of opinion that the maximum of a first dose should perhaps be two grains. (p. 271.) Sir William Ellis says little in its favour; asserting that "it more frequently creates heat and general febrile action than procures sleep." If given at all, he is of opinion that it should be in conjunction with ipecacuanha. (p. 171.)

Hyoscyamus is sometimes very useful, in full doses; but, according to Dr. Prichard, not a remedy of much importance. We have also certainly observed, in many instances, the languor and uneasiness attributed to its administration; but there are cases in which its influence is marked and salutary. Camphor has been strongly recommended, but we do not think that English practitioners have much experience of its effects in maniacal disorders. To allay the irritability of incipient insanity, and that which attends the exacerbations in old cases, is certainly a desideratum in medicine; and we wish we could entertain the consolatory belief professed by Sir William Ellis, (p. 187,) that there is in nature some undiscovered medicine which would act as a specific in these cases.

Mercury, which has been recommended for everything, has been largely employed by some physicians, in this and other countries, in cases of insanity. Such cases may occasionally depend on causes removable by this valuable remedy; and, as Dr. Prichard observes, it may be expected to be especially serviceable in cases of torpor, with defective secretions. But its general employment is, we are well convinced, very hurtful; and in some cases productive, we suspect, of great irritability of the brain.

That there are cases of mental disorder in which tonic medicines and diet are of extreme importance is agreed by all who have much acquaintance with these disorders. The general opinion given of these and other medicines by M. Esquirol perhaps conveys an idea of the result to be expected. "Camphor," he says, "musk, iron, quinia, antimony, have often been employed as specifics in insanity. These medicines are useful, but of an individual utility; they succeed marvellously when we are happy enough to seize the proper indication for them; but they are dangerous and hurtful if applied to all patients." (Vol. i. p. 153.)

M. Esquirol has been, he says, quite unsuccessful in his attempts to cure madness by *magnetising* the patients: he gave the plan many trials, with different magnetisers, without advantage.

We have always entertained some doubts as to the propriety of reducing a refractory patient to obedience by putting him in a chair subjected to rapid rotatory motion; being inclined to class this device, as well as the "bath of surprise," with other species of torture formerly too common in cases of insanity, but now happily abolished. As a remedial means, however, it is impossible to deny that practical men consider it occasionally useful and advisable. It induces nausea and sickness, which have been found to put an end to paroxysms of violent excitement. It is requisite for the success of this means, that the rotatory motion should be continued until vomiting is induced; and as sickness, and violent purging, and fainting, and great prostration of strength, and sometimes epistaxis, and a threatening of apoplexy, are among its consequences, we are glad to learn from M. Esquirol that its use is abandoned in the French asylums." (Vol. i. p. 156, *note*.)

The most difficult task for the practitioner must often be to ascertain on what condition of certain organs, remote from the brain, the cerebral irritation depends; and yet this knowledge must of course in such cases precede the due employment of means of relief. In cases of long continuance, the period has generally passed in which treatment directed immediately to the condition of the brain is likely to be very serviceable, and some removable chronic disorder may exist in the chest or abdomen, and on its removal the mental affection may undergo considerable or complete alleviation. The fact, spoken of by Dr. Prichard as established, (p. 275), that many lunatics have been cured by a course of remedies adapted to the restoration of their general health is of very great practical importance. M. Esquirol says that the chances of cure in insanity, but especially in melancholia, always offer more hope when we are able to perceive some disorder in the functions of assimilative life. (Vol. i. p. 476.) In the Gloucester Lunatic Asylum attention to the general health seems to be the principal indication of cure that is regarded; not only in old cases, but in instances in which the disease is in a stage of *incubation*, and with the effect, Mr. Hitch assures Dr. Prichard, of preventing such cases passing on to actual mania. The medical treatment called for in each case in which the mental disorder depends chiefly or entirely on some disordered condition of the heart, the liver, the stomach, the intestinal canal, the uterus, &c. must of course vary in each particular case, and be conducted on general principles. It has been found that in exhausted subjects the adoption of a liberal diet is often followed by the best effects; even calming existing irritation. Various kinds of employment in the open air are also now adopted, with excellent results, in all well-regulated asylums.

Some practical observations of M. Esquirol in relation to paralytic lunatics are of much consequence to those who have the charge of them. They eat voraciously, and permit enormous quantities of food to collect in the pharynx, which sometimes they are unable to swallow; they consequently become threatened with suffocation, from which nothing but prompt aid can save them. One case is related in which the patient had several times nearly choked himself with calf's head, and at length, attempting to swallow a larger morsel than usual, could not be relieved from it, and died. The œsophagus was found greatly distended by a piece of calf's head impacted in it; the brain was very red, the membranes were thickened and injected, and the lungs were gorged with blood. (Vol. ii. p. 279.) Another circumstance which requires vigilant attention is the obstinate inactivity of their bowels; the rectum being paralysed, defecation becomes almost impossible: the bowels may remain in this state twenty or thirty days without any complaint being made by the patient; and inflammation and mortification may be the results. Purgatives and frictions of the abdomen have no effect. Sometimes it is necessary to unload the rectum by mechanical means. Retention, but more frequently incontinence of urine, are also among their afflictions: patients in the latter state require great attention to their bedding and clothing, and washings with aromatic infusions or spirit and water. Paralytic patients frequently allow themselves to be burnt by the fire, and even dangerously, without complaining: they are also liable, in endeavouring to change their position, to fall on the head, and die in a few days afterward. When these consequences have followed a fall out of bed, M. Esquirol has several times found ecchymosis of the dura mater, extending to the subjacent arachnoid; or a circumscribed sanguineous effusion, membraniform, and extended over the outer layer of the arachnoid.

So many large and even splendid asylums for insane persons are now established in several of the countries of Europe, that one would suppose there is little necessity now existing for enforcing the value of a well chosen site and a well ordered building, and dry and cheerful airing grounds. The evils which remain, in this respect, in this country, are not to be met with in the public institutions, nor even, we hope, in many of the private establishments; although some of the latter there undoubtedly still are in which a want of space and of proper ventilation, and of a sufficient number of attendants, and general arrangements for comfort and cure, are but too obvious. That the results are lamentable on the health of the unlucky inmates we feel perfectly assured; as indeed no one can doubt without doubting the justness of M. Esquirol's observation, that the constitution of lunatics has a tendency to become rapidly weaker. They contract, he says, diseases of the skin, lymphatic engorgements, and scorbutus; and these, he says, are reasons for care in the selection of the site and design of buildings for their habitation. How rapidly old-looking they become, every practitioner must have observed; and in some of the old-fashioned institutions we fear that some of those terrible examples are even now sometimes to be seen, which were formerly far more common, of the extremest bodily and mental helplessness induced by positive neglect; neglect of cleanliness, want of good air, and of sufficient clothing, and of proper food either for body or for mind.

We have thought it desirable to allude to the opinions of some of the experienced authors whose works we are now considering on the medicinal treatment of lunatics; because it includes many particulars concerning which practitioners of great judgment and candour are found to differ; and these particulars are so important as to deserve careful reflection and clinical experiment. With regard to the mental and moral treatment of the insane, it is consolatory to believe that few, if any, medical men are now unacquainted with the principles on which such delicate attempts at control and management should ever be conducted. In the section devoted to this part of the subject in Dr. Prichard's work, and in various parts of M. Esquirol's writings, the reader will find an ample exposition of all that it is most requisite to keep in mind in the attempt to perform duties of a most arduous and responsible nature. The separate questions, all of which at some time or other occasion anxiety in every practitioner's mind, of the seclusion of insane patients, of removing them from particular impressions and associations, of the extent and nature of the control to which they should be subjected, and of the management of the infirm understanding, are fully discussed by both these distinguished writers. These questions comprehend so many interesting topics that we must even at present refrain from their enumeration, strongly recommending to every student the perusal of those parts of the works which we have mentioned in which these matters are spoken with the fulness which is due to their importance. In an especial manner we would direct the reader's attention to the section on the treatment of *mania* in M. Esquirol's second volume; page 187 et seq., wherein he will find most important principles illustrated by very striking examples.

One observation, however, suggests itself very forcibly to the mind on perusing M. Esquirol's admirable remarks on the advantage arising to the medical attendant on the insane from a friendly and almost constant communication with them; as well as from several remarkable examples related by him of the effect of moral impressions made on the patients. The first idea of the treatment applicable to each case, he says, is often derived from something observed in the actions, the looks, the aspect, the conversation, or the gestures, and in shades of these which are imperceptible to others. (Vol. i. p. 116.) And again, speaking of the treatment of cases of melancholia (p. 465), he observes that the moral medicine which seeks in the heart the primary causes of the malady, the medicine—which pities, weeps, consoles, partakes the sufferings and reawakens the hope of the patient, is often preferable to all other. Every melancholic patient, he adds, (p. 472,) must be controlled by a perfect knowledge being acquired of his mind, his character, and his habits. We need not accumulate passages expressive of the same opinions: but need we remark, that to expect that medical men should, generally speaking, possess the delicate knowledge here spoken of by Esquirol, possessing, as they at present do, so few opportunities of studying these indications of the degrees and shades of disordered mind, would be visionary in the highest degree. Until opportunities are enjoyed by students of seeing lunatic patients with as much facility as other patients, it will be vain to expect much improvement in their treatment; the care of which will continue necessarily to devolve for the most part upon men of some practical acquaintance with asylums, but who have not had the preparatory education requisite for effecting improvement, or for conducting the cure of the various and delicate cases intrusted to them on enlightened principles.

When the actual responsibility of a patient affected with mania devolves upon a practitioner, the immediate difficulty which besets him is that of preventing the patient from injuring himself or those around him. Timid attendants and alarmed relatives look for prompt aid and security to the medical attendant, and he must be prepared accordingly. For want of moral courage, or other and better resources, mere force and severity are often resorted to, and the unfortunate patient is injured, and obstacles arise out of the injudicious cruelty with which he is treated which may impede eventual recovery. Firmness and kindness,—perfect firmness, and kindness not to be averted from its objects by the perverseness of the patient, will be found more powerful than threats and severity. Dr. Prichard quotes the excellent directions of M. Georget, as condensing the discursive opinions of Pinel and Esquirol on some of these points. (p. 299.) It is not to be forgotten that patients who manifest a tendency to suicide, murder, or other violence, must never be lost sight of, or trusted with the means of mischief. For these, and often for patients addicted to indecent practices, a strait-waistcoat is necessary. Sometimes it is better to shut them up in their rooms; or to confine them by straps round their legs, fastened down in an arm-chair. When the use of chains was discontinued, by the philanthropy and courage of Pinel, it was found that the number of furious lunatics was diminished, and the accidents which formerly occurred became less frequent. The strait-waistcoat, seclusion, removal from one part of the asylum to another, the shower-bath, and occasional privations, are the means found most efficacious in public institutions. It is particularly remarked by Esquirol that a violent lunatic is only the more enraged if opposed by one or two keepers; whereas if a great force is brought to bear upon him at once, or six or eight keepers are brought before him, he will desist from all violence and submit. If necessary, he may be approached and seized on all sides; or he may be bewildered by his head being suddenly enveloped in a napkin.

M. Esquirol's plan in the first period of *maniacal* insanity is to place the patient in a room freely ventilated, but admitting little light, cool if the season is hot, and warmed in cold weather. If the patient is extremely violent, he is fastened on his bed, or his movements are confined by the strait-waistcoat. A severe diet is prescribed; cold drinks, barley-water, or whey, or almond emulsion, or cherry-water, or pure water, and other simple diluents are given. The patient is left alone, the attendants being at the door, and relatives and friends being excluded, "so as to reduce the patient to the smallest possible number of impressions and excitements." But this treatment is only adapted to the first period. (Vol. ii. p. 183.) After that it is desirable, and even necessary, that they should be allowed free exercise in the open air, and sufficient scope for their harmless extravagances, to the fullest extent, indeed, compatible with their safety and that of others. Restraint too long continued increases first the fury of the patient, and secondly, the chances of supervening paralysis. The stage of convalescence requires different measures; change of place, diet, and occupations.

Among the various methods of restraining the violent, particularly in the first stage of the malady, that mentioned by Sir William Ellis seems to possess several advantages.

"The most simple and least objectionable mode of confinement, is that of a pair of wide canvass sleeves, connected by a broad canvass shoulder-strap, so as to rest easily on the shoulders. They ought to come up well on the shoulders, and to extend about an inch beyond the ends of the fingers: the part covering the hand should be made of tolerably stiff leather, to prevent the hand grasping anything. They keep the arms hanging easily, and in a natural position, by the sides of the body. They are fastened at the back by two straps, one going from one sleeve a little above the elbow, across the loins to a similar position in the other sleeve; a second lower down, and by three similar straps in the front, the latter being secured by buckles, which, in large establishments, where there are many patients to be attended to by one keeper, ought to be locked. This mode of fastening has many advantages over the strait-waistcoat. In the first place, it is less heating, it produces no pressure upon the chest, and the arms, though secured from mischief, have so much freedom that the blood can circulate freely; as with these sleeves ligatures of every description are unnecessary. It is sometimes also requisite to secure the feet. For this purpose we find, that a couple of leathern straps well lined with wool, placed round the ankles, and secured to the bed by staples, is all that is necessary. In hospital practice cases will sometimes occur, where it may be necessary to secure the bedding in its place. This can be done by having a thick quilt fastened over the blankets, by three leathern straps, to the sides of the bed. It occasionally happens, that, unless this precaution is taken, the patient will toss all the clothes off the bed." (p. 164.)

In some cases we have seen that M. Esquirol mentions the fatal results of a refusal to take food. It is probably found, in the course of the extensive practice afforded by a large asylum, that this case presents more difficulties than would at first sight appear to attend it. Doubtless in many cases it is possible to introduce food into the mouth of the patient by closing the nostrils, in the manner so successfully adopted when the object is to force children to swallow unpalatable medicines; and the patient has so great an aversion to this process, that its repetition is not always necessary. This method seems at least as easy to practise as it can be to introduce the tube of the stomach-pump, in the manner recommended by Mr. Liddell, the translator of M. Esquirol's work on the Illusions of the Insane. As the repugnance to take food often depends on the supposition of the patients that it is intended to poison them; this delusion should, if possible, be combated, or the employment of force may produce hurtful agitation. As it not unfrequently is associated with the belief that instead of food they see pins, needles, or nauseous and disgusting substances presented to them, it is possible that some of the patients might be persuaded to take food when blindfolded. These cases often require forcible and yet judicious appeals to the feelings of the patients. If all means fail, M. Esquirol advises the introduction of a tube through the nostrils: by this means he has saved many lives: in a case recited (Vol. i. p. 663,) it required to be used nearly five months.

These measures are chiefly useful in the melancholicpatients and those who intend to destroy themselves by starvation. Maniacal patients require, M. Esquirol maintains, a different treatment. In these cases the repugnance to take food does not often continue many days. Sometimes it depends on temporary disorder of the stomach, and sometimes on a degree of delirium which takes away all sense of want: in the latter instances, a blister applied to each leg sometimes overcomes the refusal to eat.

Compelled, as we are, by the extent and number of the subjects comprehended in the general consideration of insanity, to abstain, at least for the present, from entering upon the important subject of moral treatment; we cannot refrain from recommending to the philanthropic consideration of all medical men the importance of houses of retreat and separation from lunatics of the *convalescent*. Without such a retreat, the cure must often be imperfect; many unhappy patients must suffer relapses, and many linger long in a state of uncertain recovery, to the great detriment of their worldly affairs, to the great misery of their families, and to the utter ruin of their own happiness. For patients of the comfortable classes, it is easy to provide every advantage which their condition requires; but for the poorer classes of society, whose affairs are often thrown into disorder by their unfortunate malady, it is far more difficult to provide a suitable convalescent abode. Sir William Ellis comments on this subject with equal good sense and humanity.

"Unfortunately, when a poor man, who has been for a long time an inmate of a lunatic asylum, where his daily wants have been supplied without any care or anxiety on his part, becomes sane, there is great difficulty in introducing him again into the world, and making him entirely dependent upon his own exertions, without at the same time producing a greater feeling of anxiety than his enfeebled brain and nervous system are capable of bearing. Many of the paupers, on their recovery, are entirely without resources; and they are driven of necessity into the workhouses, until they can obtain employment: this is more than they are able to bear. The benevolence of a gentleman of the name of Harrison, has done much to relieve cases of this kind, occurring in the West Riding of Yorkshire. Her Majesty Queen Adelaide is the patroness of a charity, which has for its object the supply to the immediate and most pressing necessities of the paupers, when discharged cured, from the asylum at Hanwell. Her Majesty contributed to it one hundred pounds, and other sums have already been subscribed, which have raised the amount of Queen Adelaide's Fund to the sum of nearly one thousand eight hundred pounds: this has been invested in the funds; and the dividends have, in several instances, been the means of affording such timely assistance, as has, in all probability, prevented a relapse, and enabled the convalescent to maintain himself in comfort and respectability.

"But something further is still wanted. A comfortable place, where such of the patients as might be deemed proper objects, might, for a time, find food and shelter, and a home, until they could procure employment, would be an invaluable blessing to them; and if such an institution were established, even at the cost of parishes, it would in the end prove a saving. Many patients might be tried in such an establishment, and eventually restored to society, who are now compelled to remain in the asylum as lunatics, in consequence of their retaining some erroneous view, on some unimportant matter. Although this does not interfere with their capability of judging between right and wrong, or prevent them from performing their duty, it is an insurmountable bar to a medical superintendent signing a certificate of their sanity; and without this, the visiting justices cannot order their discharge. I have no doubt, that in many instances, this erroneous impression would be effaced by a little mixing in the world, and in the ordinary business of life: indeed, I have known cases of this kind, where the friends have made the trial, and have procured the discharges of the patients, on their undertaking that they shall be no longer a burden to the parish. The greatest success has been the result: the complete change of scene, and the occupation of mind have entirely diverted the thoughts from the subject on which the erroneous impression remained; and as this ceased to be dwelt upon, the derangement gradually wore off, and the patient soon became perfectly sane." (p. 250.)

The portions of Dr. Prichard's work devoted to the Statistics of Insanity; to Unsoundness of Mind in relation to Jurisprudence; to Ecstatic Affections, including the phenomena of Somnambulism and Animal Magnetism; we can only mention the titles of. Much information is conveyed in each. A supplementary note of twenty-two pages contains the objections of this eminent physician to phrenology; on which we shall make no further comment than to observe, that, admitting the justness of Dr. P.'s remarks, we do not think them subversive of the phrenological doctrines. The work, taken altogether, is one of the most valuable books on insanity in our language, and one which every practitioner should make himself well acquainted with.

In like manner, there are large and valuable parts of M. Esquirol's volumes which we do not design to make the subject of present observation. Such are the section on Suicide, in the first volume; and the Statistical and Hygienic Memoirs, and the section on Mental Alienation in regard to Legal Medicine, which occupy more than half of the second. In these will be found, by those who carefully consult them, many valuable results of M. Esquirol's long observation; including many notices bearing upon practical points of much interest to those who have the care of lunatics. The Atlas of Plates contains twenty-five most expressive delineations of the several forms of insanity; particularly of melancholia, mania, and of cases passing or passed into dementia. The history of the individuals of whom the plates represent the likenesses are very interesting. That of Théroigne de Méricour, a highly-gifted woman, but celebrated for her irregularities and her political activity during the revolution, is a most impressive narrative of attractions and powers misapplied; and we can scarcely imagine anything more touching than the fixed and hopeless expression of the lady who is the subject of the third plate; who, having been in childhood the playmate of the unfortunate Duke d'Enghien at Chantilly, became, on his death, when she was but seventeen years of age, suddenly grey; sunk into profound melancholy, and remained almost wholly motionless and silent, her eyes for ever fixed on the window where she seemed to hear or see some one who engaged her attention; until death put an end to her incurable sorrow. These, doubtless, are but a few of the dreadful commentaries, afforded by the madhouse, on the crimes of those reputed sane.

Dr. Allen's Essay on the Classification of the Insane promises, by its title, to treat of a very important subject; but the classification of the subjects in the work itself is extremely defective. Both in this work and in Sir William Ellis's, but most in Dr. Allen's, the rambling style is such, and the plan so unintelligible, that the works have, properly speaking, neither beginning, middle, nor end. Dr. Allen accounts in part for the oddities of his production by saying "it was written, and even a great part of it printed," as a continuation of his defence in the case of Allen v. Dutton, of which defence, we regret to say, we have no recollection. The preface promises that the said defence shall be bound up at the end of the essay, but we have looked for it in vain. Anything less consecutive and distinct than the preface we have not for a long time perused. To this succeeds what is called an Essay on Classification, extending to 110 pages; and to this is joined an appendix of 102 pages, with ten portraits of lunatics. The Essay begins with an account of Fair Mead House, Leopard's Hill Lodge, and Springfield, which constitute Dr. Allen's establishment at High Beach; all these localities, we fear, being not much known to readers in general, although we have little doubt that they furnish comfortable and well-conducted retreats for the insane. The arrangement of them is made with the very laudable design of classifying the patients, and particularly of subjecting the "recent, partial, slight, or convalescent cases" to particular attention. A remarkable case is introduced, abruptly enough, but professedly, as the table of contents says, to illustrate this subject; which case does indeed illustrate Dr. Allen's judicious management of a most violent and miserable fanatic, but has not the least bearing on the subject of classification. Every successive page convinces us the more of our incapacity to fathom the scope and tendency of Dr. Allen's meaning; and yet the detached observations, detailed in a kind of colloquial, reminiscent manner, are often sensible enough. The theory, however, dispersed here and there, is not quite so much approved of by us; we demur to "an alteration in the state of the nervous energy, *generating an acrid and morbific matter in the system*, and ultimately disease." (p. 22.) At page 55, we have a whole page of the preface, most unaccountably thrust into the middle of the essay, not transposed by any sleepiness of the printer, but repeated, as it would appear, by an oversight; and this tends to explain the strangeness both of Essay and Preface. The remainder of the essay consists of cases and observations, neither of which present any kind of novelty, although some of the cases are curious, and the practical observations, generally, are unexceptionable. The appendix contains thirty-one cases; some of which are instructive, and some highly amusing. We are bound to confess that the heads represented in the plates furnish very respectable phrenological testimony.

Of the benevolent views of Sir William Ellis, and of his great practical experience, his Treatise gives sufficient proof. The description which it contains of the large institution at Hanwell would alone render it valuable to medical readers. Its style, as we have already observed, is discursive, and we do not see much in the chapters on the nature, symptoms, and causes of insanity, with which previous works had left us unacquainted. We cannot but think, also, that Sir William Ellis lays too much claim to the merit of employing the lunatics; both because this system is not now to be considered a new one, and because when so many inmates of an asylum in which the cures are so few in number, and the incurable so numerous, are exhibited to the public as affording a spectacle of tranquil and useful occupation, a suspicion intrudes itself that some, at least, of these individuals who are classed among the incurable, might be restored to their ordinary modes of life; which result, and not the mere *house-keeping* of an asylum, much less its vain-glorious display, should surely be the first object of the physician's ambition. At present, our lunatic asylums, or some of them at least, exhibit in their management a singular anomaly; for whilst the student, to whom a familiarity with their economy, and with the changeful and striking phenomena which their inmates present, would be extremely useful, is sturdily denied all entrance, as if his intrusion was a thing not to be endured, the public at large throng to these places on Sundays and holidays as to a fair. The careful, anxious, zealous student might sometimes be of service, and in many modes and on many occasions an auxiliary of the physician in chief; but the miscellaneous visitors must often be mischievous. Sir William Ellis, when speaking of admitting pupils to Hanwell, evidently feels that he is treading on unsure ground; and yet Hanwell is one of the *sights* of the vicinity of London. This publicity, if in one sense useful, inasmuch as it is a security to the public that all is well conducted there, is to us a far less noble recommendation than would be the circumstance of that large institution being at once the best conducted *hospital* for madness, and the spot on which a number of diligent students were ever imbibing the principles of rational treatment, to be by them diffused over the whole kingdom, or over the whole world. This would be an object worthy of the aspirations of the governors, and worthy of their philanthropy; it would act as a stimulus of the most salutary kind on the medical officers; and be the source, we really believe, of incalculable benefits to society. Then we might expect to receive, from time to time, from that establishment, with the weight of authority which a wide field of experience always gives, statistical details of the highest value, new and varied pscychological and pathological observations, and clinical results by which this department of medicine would be raised from its present very unsatisfactory condition. We might then, in short, have something to point to, worthy of comparison with the writings of Esquirol and Foville, of Guislain and of Bayle.

Among the works of which the titles are prefixed to the present article, is one by Dr. Crowther, of Wakefield, a physician whose practical acquaintance with establishments for lunatics and great respectability entitle his remarks to much attention. He takes up his pen, at a time of life in which the favour or disfavour of men is of little consequence, as the advocate of the insane, with the intention of fearlessly pointing out the evils to which they are even yet, in his opinion, exposed. Although we do not apprehend, as Dr. Crowther appears to do, that any want of public vigilance could ever cause "the evil genius" which "so long tenanted Bethlem" to "domicile itself in Hanwell or Wakefield," we are much impressed with the force of many of his observations on the government of institutions of this kind. The difficulties in this particular of government are numerous; and it is more easy to point out some which arise from the dominion of the magistrates than to suggest safer and more efficient hands to which to intrust the power which must somewhere exist. We fear Dr. Crowther's remedy would be neither pleasant nor efficacious. The very name of a medical board is synonymous with discords, intrigues, mutual accusations, and every variety of strife that can render science ridiculous and impede the progress of truth. Magistrates may, and do, frequently err, for they are but mortals; but, where no political or religious prejudices interfere, we would rather trust to their plain integrity and sound good feeling than to the refinements of any association of men of a scientific description. It sometimes happens, no doubt, as Dr. Crowther observes, that magistrates make very unfit appointments, and that they are inclined to overlook delinquencies in the instruments they have unwisely selected; but a medical board would be equally fallible. The medical superintendent of a lunatic asylum may occasionally be thwarted, and have his patience considerably tried by an officious magistrate; but among several magistrates he cannot fail to find some men of sense who will support him in all that deserves support. Unhappy beyond all functionaries would he be who had to listen to the opinions, and be driven to and fro by the fancies of half a dozen men of his own profession; each " jealous of honour, sudden and quick in quarrel." In the one case, good measures would sometimes be delayed; in the other they would much more frequently be frustrated. The physician who acts with magistrates is surely pretty safe, if he exhibits ordinary capacity and judgment, from interference in his medical management of the insane; which, it is too often forgotten, is the most important point of his duty: but he who acted under a medical board could not prescribe bleeding or a purgative without fear of censure or impeachment. The magistrates may be omnipotent in the board-room, in the larder, the dairy, the kitchen, and the laundry; but they refrain from the doctor's shop. The medical board would be everywhere. Our opinions on these matters are formed after some experience of institutions governed by both classes of men, and we entertain a strong impression that no men are less fitted to govern others than those whose minds are narrowed by exclusive devotion to science. On this point we find ourselves completely at issue with Dr. Crowther; and his opinion would lead us much to question the soundness of our own, if we did not believe that he speaks rather from experience of one of the kinds of government of which we have spoken than of both. Perhaps the best government of all medical charities and institutions is that by a board or committee, of which a certain portion consists of medical men; but the portion ought not to preponderate.

On another point this venerable physician has, however, our entire assent. We mean, as respects the unlikelihood of the treatment of insane patients being well conducted, with a vigilant and unprejudiced eye towards the improvements gradually arising from advancing science, by any one whose mind has been directed to the management of mental diseases only. Dr. Crowther's observations on this subject are most judicious, and abundantly show the delusion of looking upon the cure of madness as a kind of craft, in which men wholly ignorant of medicine can become proficients. The third chapter of Dr. Crowther's little work, *On Quackery*, has our hearty approbation; and we concur with him in opinion that the diffusion of knowledge will alone efficiently protect regular medical men from " watering-place doctors," of whose proceedings he seems to have a very intimate knowledge. The fourth chapter relàtes to the prevalence of dysentery in asylums, which, especially at Hanwell, and in the West Riding and Kent asylums, Dr. Crowther ascribes to negligence. The sixth chapter relates to certain alleged fallacies in the returns of cures in the lunatic asylums; and especially at Hanwell; the patients who are discharged by the desire of their friends being, it is said, put down as cured. We can scarcely suppose this loose practice to exist at present. Dr. Crowther naturally enough ridicules Miss Martineau's account of the cures at Hanwell being, of the recent cases, "ninety in a hundred;" the truth being that the cures are fewer there, as we have already had occasion to mention, than in most of the asylums in the kingdom. We do not feel disposed to make particular allusion to certain other imputations of delusive reporting, from which, however, we should think Sir William Ellis would be anxious to clear himself. In consequence of reasons which actuate us in refraining from remarking on these points, we have passed over, in our notice of Sir W. Ellis's book, several observations, the tenor of which we by no means approve; and which give the work too much the air of an elaborate advertisement; partly of Hanwell, partly of another institution then in embryo; and partly of Sir William and Lady Ellis in all institutions wherever situated. Something of this kind we see also in Dr. Allen's book: and, although we entertain not the smallest doubt of the intelligence, kindness, and great respectability of Mrs. Allen and of Lady Ellis, the encomiastic leaning to the feminine head assuredly shows the ascendancy of the housekeeping department, of the propriety of which we are extremely sceptical.

The impropriety of allowing the medical superintendent of a large asylum to be the steward of the institution is very properly commented upon by Dr. Crowther. We have not the least doubt that in these cases, the housekeeping is the part of the direction best attended to. The asylum becomes a good boarding-house, a safe prison, a kind of show-house, but not an hospital, not a place of cure. That these circumstances have never been properly brought before the attention of those who appoint the chief officers of such establishments, and that they do not entertain just notions of the importance of the medical treatment of the insane, we have very good grounds for believing. We trust Dr. Crowther's remarks on the general constitution of asylums, and on the duties of the officers and servants, may attract general attention.

Dr. Ferrarese, of Naples, in the work which we have once or twice mentioned in our previous observations, appears to be a physician of much learning and of a sound judgment. We have read his work with much satisfaction; not so much on account of any particular originality in his observations, or novelty in his views, as because we have observed that he is well acquainted with the English writers, down to Haslam, with the French, inclusive of Esquirol and Georget, and with the best German authors on the subject of insanity. From these and other respectable authorities he has rather compiled a well-digested work than composed a treatise on disorders of the mind. But in every part of the work we perceive uniform indications of good sense, of enlightened views, and of benevolent and well considered plans of treatment. The work forms, indeed, an excellent epitome of theory and practice in the various forms of insanity.

The Tenth Report of the Connecticut Retreat comprehends a period of three years. About one hundred patients are accommodated in the institution. The recoveries are stated as being equivalent to about thirty per cent. in the old cases, and about ninety in recent cases.

About one hundred patients are generally lodged in the Lunatic Asylum of St. Petersburgh. Of those admitted in 1834, eighty-eight in number, six were received for the second time, and three for the third time. The majority of the male patients were unmarried, and of the women, married. The greater number were admitted between the thirtieth and thirty-fifth year. Of those discharged cured and convalescent, the following were their periods of staying in the house:

| | | | |
|---|---|---|---|
| In the first month | 5 | In the eighth month | 4 |
| — second | 4 | — eleventh | 6 |
| — third | 5 | In two years | 2 |
| — fourth | 3 | three | 1 |
| — fifth | 3 | six | 1 |
| — sixth | 5 | | |
| | | | 14 |
| | 25 | | 25 |
| | | Total | 39 |

In 1833, Dr. Greco, physician to the Royal Institution for the Insane at Palermo, published a pamphlet on the medical statistics of that institution from its reformation, about thirty years previously, in consequence of the humane efforts of the Baron Pisani. In a second edition, Dr. Greco has completed his tables up to 1835. On these we can only make a few passing observations. It is remarkable that the number of women admitted is less than half that of the men. In the St. Petersburgh report the number of men and women is nearly equal. In ten years at the Connecticut Retreat the number of men and women admitted is exactly the same. We have already mentioned Esquirol's researches on this point. But a question presents itself here which materially affects the value of hospital statements in a statistical point of view. The admissions of men and women, it is evident, may often depend on the accommodations and regulations of the hospital; and afford no correct evidence of the proportion of men and women affected with insanity in the neighbourhood. In the Dundee asylum, the admissions (18th Report) are twelve women to thirty men; and there the cause is stated to be, as might be expected, a want of more accommodation for female patients. Of 508 cases admitted into the Retreat at York, 245 were men and 263 women.

Idiocy seems rare in the fine climate of Sicily; giving support to the opinion of Esquirol that whilst insanity increases with the progress of what is called civilization, idiocy depends more on the influences of soil and climate. This conclusion, however, we should by no means consider as established: the vices of civilization will probably be found to be as productive of idiocy in the offspring as any climatorial influences. We derive salutary information from the prevalence of insanity in commercial communities; but it is not equally known, we think, how much the somewhat solitary, unexciting, indulgent life of the agriculturist disposes to mental disorder; nor has its singular frequency in the chief houses of some mountainous districts of our own island been the subject of much observation, although well known to tourists. We suspect, also, that the number of cases closely allied to imbecility of mind is very great among young persons composing the aristocracy of our quietest country-towns, and in the monotonous country-houses of England ; these cases escaping general observation, because such minds are called upon for little or no exertion; but the medical observer cannot overlook them. Talents little cultivated and little exercised for several generations, and a mode of life almost entirely animal, are circumstances which lead to the production of a robust fine-looking progeny, often with large round heads, but with a brain of the smallest degree of activity. Even the phrenologists have failed to notice these curious particulars, which our small provincial communities, in parts of the country unenlivened by extensive trade and railroads, afford, we think, some opportunities for observing.

Dr. Greco introduces a table of *occupations,* which we regard as very interesting.

| MEN. | | WOMEN. | |
|---|---|---|---|
| Merchants | 14 | Servants | 25 |
| Military men | 27 | Peasants | 20 |
| Naval men | 2 | Tradespeople | 36 |
| Priests and monks | 27 | Nobility | 9 |
| Students | 9 | Private gentlewomen | 39 |
| Medical men | 4 | Nuns | 6 |
| Lawyers | 16 | Poor people | 17 |
| Nobles | 6 | | |
| Squires living upon their estates | 20 | Total | 152 |
| Placemen | 23 | | |
| Industrious civilians | 30 | | |
| Peasants | 44 | | |
| Servants | 3 | | |
| Artisans | 55 | | |
| Beggars | 3 | | |
| Public salesmen | 13 | | |
| Prisoners | 10 | | |
| Total | 306 | | |

In this table we are anxious to point out to the reader the number of cases of insanity among the poorer classes of society; because we do not think the influence of poverty and wretchedness has been sufficiently considered in relation to the imperfection and disturbance of reason. We see that, among the male cases, the peasants and artisans are the most numerous; and if to these we add the servants, the beggars, and the prisoners, we have more than one third of the male lunatics in the humbler ranks of society. Among the women, leaving out the tradespeople, the number of peasants, servants, and poor people is nearly one half of the whole. Other reflections, on which we need not dwell, will arise in every reader's mind on looking at the table; and their relation to the causes, and consequently to the prevention of insanity, is not unimportant. We should observe, that the Sicilian peasant is not only exposed to poverty, but to the burning rays of the sun, and to the vapours of charcoal and metals. The moral causes of insanity are many in Dr. Greco's tables. One half of the cases of this kind are ascribed to domestic sorrow, destitution, reverses of fortune, ambition, the loss of parents, and persecution. In the Aberdeen Report (1838), of forty-three patients admitted, the causes of the malady were, intemperance in the use of spirits in eight, domestic disquietude in seven, pecuniary difficulties in two; and, in twenty-two out of the forty-three cases, there was also hereditary predisposition. In the Massachusetts Hospital, the proportion of mechanics is considerable; and the reason assigned is their sedentary mode of life. Mercantile speculations are everywhere an acknowledged cause of great frequency; and it is observed by Dr. Woodward that very few of the steady, industrious, and temperate members of the community become insane. The more we consider these matters, the more clearly we see that the physician is in a great measure the substitute of human reason and of political wisdom; and that, as these are more and more cultivated and improved, he will be less and less required to interfere.

Every Report contains gratifying evidence of the curability of recent cases of insanity; and, making every reasonable deduction from these statements, sufficient encouragement is held out by them to prompt treatment, of which the neglect is so often seen to lead to the chronic and unmanageable forms of mental disorder. The Report of the New-York Hospital gives 98 per cent. as the number of recoveries in the recent cases. In this, and indeed in the various Reports which we have seen from other countries, we observe, with much satisfaction, the general diffusion of rational views of moral and physical treatment. Nowhere do we see this more distinctly than in the Scotch Reports, in which the characteristic energy of our northern neighbours is very advantageously exhibited. Accurate numerical statements are accompanied by reflections full of good sense, and entitled to be called philosophical. Everywhere, we trust, (at least in the more advanced countries of Europe,) may it now be said, as in the Dundee Report for 1837-38;

> "Gentleness, candour, exercise, and useful labour have now succeeded to harshness, deception, inactivity, and rest. The hands that were formerly bound in chains are now handling the spade and the mattock; the mind that was once bewildered by the arts of false misrepresentation, is now soothed by the voice of truth; the solitude and rest which once benumbed the faculties, and rendered torpid all our energies, are now exchanged for the employment of social life; and the heart once sunk in the gloom of religious melancholy, is now gladdened by the sound of praise and the tidings of salvation."

The concluding words of this quotation contain an allusion to a subject of great seriousness and importance, the administering of religious consolation to lunatics; than which nothing requires that zeal should be more carefully tempered by judgment.

The healthiness of the Dundee Asylum has been remarkable: its inmates wholly escaped the cholera, which was so fatal in many lunatic establishments. The average of deaths for six years past has not been more than six per cent. Upon the whole, we have been extremely interested with the reports of this admirably conducted asylum; for such we cannot doubt its being from the very nature of the reports, in which, without ostentation or any apparently interested or *business* motive, the gentle, considerate, and scientific character of the management is very conspicuous.

Among the pamphlets before us is one of the Reports of the Retreat at York, an institution which we never see mentioned by any writer on these subjects without expressions of admiration and respect. This report is for 1836, but comprehends statistical statements founded on the experience of forty years in that establishment. Most of these have already been alluded to.

It has been with much gratification that we have gone through the Reports and other documents relating to the State Lunatic Hospital at Worcester, Massachusetts; from which might be selected striking illustrations of the former dreadful condition of lunatics, and of the advantages derived from a change of treatment. The American physicians keep pace with the most enlightened practitioners of Europe in their treatment of insanity, and, whilst alive to the importance of moral management, lay proper stress on the benefit to be in many cases derived from the timely and judicious application of medicinal means. In one of the Reports it is stated that of the cases of insanity from intemperance, about 50 per cent. recover; of those from domestic afflictions, about 53 per cent.; of those from ill health, about 72; and of those from religious causes, about 50. Of the cases ascribed to manustupratio, which is frequently mentioned as a cause by the American writers, only about 7 per cent. are said to recover their reason. Some frightful cases of homicidal insanity, intended to illustrate moral insanity, are inserted in an appendix. It would be injustice to Dr. Woodward not to mention that his indefatigable attentions, philanthropy, and intelligence, in carrying into full effect all means of cure in this hospital, are spoken of by the directors as "above all praise, and beyond all price."

In the Report of the Hanwell Asylum (January, 1838,) we see little on which we are disposed to make any observations. Compared with most of the reports of other institutions, it is meager, and contains little information. Sir William Ellis, however, states that the per centage of deaths at the Middlesex Asylum, from its first opening to that time, is "much smaller than in any other large institution similarly circumstanced," with which he is acquainted; and he illustrates this by a table of the number of patients and deaths in thè Middlesex, Wakefield, and Lancaster Asylums; the only three large asylums in England, he says, in which *only* paupers are received, and "where the patients must remain until they die or are cured, or cease to require parochial assistance." By this table, whilst the annual per centage of deaths for six years past has been, at Lancaster 24.29, and at Wakefield 17.87, it is shown to have been only 12.56 at Hanwell. This is chiefly ascribed by Sir W. Ellis to the good situation of the Hanwell Asylum; but as, of nearly 600 patients remaining in the asylum at the time of making the report, Sir W. Ellis pronounces all to be incurable, at least in all probability, *except thirteen*, the rest must of course die off, and swell the annual mortality in future. Of thirty-seven men admitted last year, fourteen were discharged cured; and, of twenty-seven women admitted, the cures were thirteen. The chronic cases, or those of longer date than two years, were, of the men, seventeen, and of the women, eighteen. Of the rest, six men and two women had been previously insane. A great number of the patients (194 men and 260 women) are constantly employed. We cannot help again expressing something like a belief, or certainly a hope, that some of these patients, so docile, so industrious, so orderly, may yet be restored to perfect mental health by various, well-devised, and timely applied medicinal and moral means.

The brief notices we have already given of Mr. Farr's Statistics are sufficient to show the variety and importance of the particulars included in his pamphlet. On one subject, which greatly requires revision, the *public management of lunatics*, we cannot at present enter. The present system is certainly neither convenient nor effective. In Mr. Farr's words,

"At present there are inspectors for Chancery lunatics, commissioners for licensed houses, visiting justices for county asylums: Bethlem, where the greatest abuses have existed, is expressly exempted from regular inspection; the reports of the best asylums, those supported by subscription, are published in no regular form; the facts collected by the central commissioners, and the inspectors of Chancery lunatics, have never been published. A more delusive, inefficient, unsatisfactory system of inspection than now obtains cannot be imagined." (p. 37.)

The remedies proposed by Mr. Farr are, that the medical inspection should be vested in one body, and should extend to all establishments irrespectively; and that every lunatic in the country should be included in the annual tabular reports. We believe many existing evils would be put an end to by this plan, the details of which will be found in Mr. Farr's pages; and there can be no doubt that it would be the means of obtaining statistical results that might be relied upon.

In the past history of lunatic asylums, revolting as some of its early passages are, the progressive improvement of those melancholy receptacles for the worst forms of human wretchedness affords a foundation for the most confident hope that whatever evils yet exist in their government will gradually be remedied. To some large institutions we understand that well-educated medical men have recently been appointed as superintendents; men who will not consider themselves as mere bailiffs or stewards, or keepers, or show-men of lunatics; and who will have little opportunity of making a public institution merely subsidiary to avaricious private views; but will remember what is expected from them, and acquit themselves of important duties to the public, as well as to the establishments to which they belong.

The preceding observations suffice, we think, to convince every medical reader that there are yet many unsettled questions to which men of scientific education might usefully apply themselves, in our large lunatic institutions. A fuller and closer investigation of the predisposing and exciting causes of insanity,—a careful investigation and trial of different modes of treatment,—and some questions in medical jurisprudence, may be particularly mentioned, as soliciting the attention of those who are enabled by their position to devote all their time to this great branch of practice; and not many years will elapse, we venture to prophesy, before valuable contributions will be made in our own country towards a more satisfactory pathology and practice in all diseases of the mind.

